# *Streptococcus pneumoniae* serotype 19A in Latin America and the Caribbean: a systematic review and meta-analysis, 1990–2010

**DOI:** 10.1186/1471-2334-12-124

**Published:** 2012-05-28

**Authors:** Elizabeth Castañeda, Clara Inés Agudelo, Rodrigo De Antonio, Diego Rosselli, Claudia Calderón, Eduardo Ortega-Barria, Rómulo E Colindres

**Affiliations:** 1 Instituto Nacional de Salud Bogotá, Bogotá, Colombia; 2 GlaxoSmithKline Biologicals, Panama City, Panama; 3 Department of Clinical Epidemiology and Biostatistics, Universidad Javeriana Medical School, Bogotá, Colombia; 4 Independent investigator, Bogotá, Colombia; 5 GlaxoSmithKline Biologicals, Rio de Janeiro, Brazil; 6 GlaxoSmithKline Biologicals, Wavre, Belgium

**Keywords:** *Streptococcus pneumoniae* serotype 19A, Latin American and the Caribbean, Resistance to penicillin, Conjugate vaccines, Serotype replacement

## Abstract

**Background:**

Pneumococcal conjugate vaccines (PCVs) are in the process of implementation in Latin America. Experience in developed countries has shown that they reduce the incidence of invasive and non-invasive disease. However, there is evidence that the introduction of PCVs in universal mass vaccination programs, combined with inappropriate and extensive use of antibiotics, could be associated to changes in non-PCV serotypes, including serotype 19A. We conducted a systematic review to determine the distribution of serotype 19A, burden of pneumococcal disease and antibiotic resistance in the region.

**Methods:**

We performed a systematic review of serotype 19A data from observational and randomized clinical studies in the region, conducted between 1990 and 2010, for children under 6 years. Pooled prevalence estimates from surveillance activities with confidence intervals were calculated.

**Results:**

We included 100 studies in 22 countries and extracted data from 63. These data reported 19733 serotyped invasive pneumococcal isolates, 3.8% of which were serotype 19A. Serotype 19A isolates were responsible for 2.4% acute otitis media episodes, and accounted for 4.1% and 4.4% of 4,380 nasopharyngeal isolates from healthy children and in hospital-based/sick children, respectively. This serotype was stable over the twenty years of surveillance in the region. A total of 53.7% Spn19A isolates from meningitis cases and only 14% from non meningitis were resistant to penicillin.

**Conclusions:**

Before widespread PCV implementation in this region, serotype 19A was responsible for a relatively small number of pneumococcal disease cases. With increased use of PCVs and a greater number of serotypes included, monitoring *S. pneumoniae* serotype distribution will be essential for understanding the epidemiology of pneumococcal disease.

## Background

*Streptococcus pneumoniae* causes invasive pneumococcal disease (IPD), which is often life-threatening in children less than 2 years old, adults older than 65 years old and immunocompromised individuals [1]. Pneumococcus is also the most common cause of bacterial acute otitis media (AOM) and sinusitis in children [1-3].

The most important virulence factor in pneumococcus is the polysaccharide capsule, which forms the basis for serotyping and vaccine formulation; 93 distinct serotypes have been identified (with the recent inclusion of serotypes 6C, 6D and 11E) [3-6]. Worldwide, 20 serotypes account for more than 80% of IPD, although their prevalence varies by region [4]. A small number of pneumococcal resistant clones, serotypes 14, 23F, 6B, 6A, 9V, 15A, 19F and 19A, have spread and display particularly high rates of penicillin non susceptibility (PNSP) as well as multiresistance profiles (MDR) [7].

Since the introduction of pneumococcal conjugate vaccines (PCVs), numerous studies have been published on their safety, immunogenicity and efficacy, in particular for the heptavalent vaccine (PCV7), introduced in the USA in 2000 [8,9]. Studies conducted after PCV7 introduction, have shown dramatic and sustained decreases in vaccine type (VT) IPD rates, carriage and herd effects [10-13]. These positive findings were followed by reports of IPD caused by non vaccine types (NVT) *S. pneumoniae* PNSP and MDR [14-18]. NVT have also been described as agents of non invasive disease [15] and nasopharyngeal carriage [12]. Data from both North America and Europe have shown *S. pneumoniae* serotype 19A (Spn19A) to be the most prevalent serotype, associated with increasing rates of MDR [19]. Consequently, attention has focused on Spn19A, its prevalence and the numerous factors leading to this increase, and how best to control its impact [20-22].

This review summarizes the available published and unpublished Latin American and Caribbean (LAC) data from 1990 through 2010 describing the prevalence and burden of Spn19A in children less than 6 years. For comparison, we also analyzed the data for the most prevalent serotypes in this region [23].

## Methods

We searched for data collected between January 1990 and July 2010 following PRISMA guidelines. Using both refined search strategies and broad spectrum, low specificity, searches (i.e. “*Streptococcus pneumoniae*” OR “pneumococcus” anywhere in the text), we reviewed all references on *S. pneumoniae* that were geographically linked to LAC countries, with no language restrictions; the targeted age group was children 6 years-old or younger. We searched the following databases: Medline (PubMed), Embase, Latin American and Caribbean Health Sciences Information (LILACS), Scientific Electronic Library on Line (SciELo) and SCOPUS. Search terms used are shown in Additional file 1a. Abstracts of recent meetings on infectious diseases were also included.

Serotype distribution data were extracted by five reviewers for IPD, non-IPD and nasopharyngeal carriage. In addition, data on Spn19A penicillin susceptibility, pneumococcal disease prevalence and/or incidence, mortality rate, and pneumococcal vaccine potential impact were collected when available. For calculation of impact using SIREVA data we assumed serotype 6A/6B protection for PCV7 and PCV10 [4].

In order to avoid duplicate data, numbers were only added from the databases of the SIREVA Project (only for invasive isolates) available via the PAHO website, [23]. Data included in the analysis: 2000–2005, 2006, 2007, 2008 and 2009, SIREVA corresponded to available information in the original sources published according to the methodology used for this systematic review.

We limited selection bias by reducing the heterogeneity of samples; most data were from the SIREVA network for invasive isolates with standardized laboratory surveillance techniques and expanded availability of protocols. For the inclusion and exclusion criteria used to select publications reporting non IPD studies, we reviewed international criteria and internationally defined and accepted sample collection and laboratory techniques. For the purposes of this systematic review, we adopted the definitions presented in Additional file 1b.

We analyzed and presented our results following standard guidelines. Prevalence estimates were computed using the number of Spn19A isolates as the numerator and the total number of *S. pneumoniae* reported as denominator, for each study. Two techniques were used to calculate the pooled prevalence estimates: Mantel-Haenszel (fixed-effects model) and DerSimonian-Laird (random-effects model) [24,25].

For invasive disease meta-analysis we only included publications reporting non- SIREVA data, considering that SIREVA data represent 96.7% of samples analyzed and would bias the pool estimation.

Lastly, we estimated Spn19A specific IPD incidence by multiplying the serotype distribution by the reported incidences identified in this review.

## Results

### Study selection

**Figure 1:**
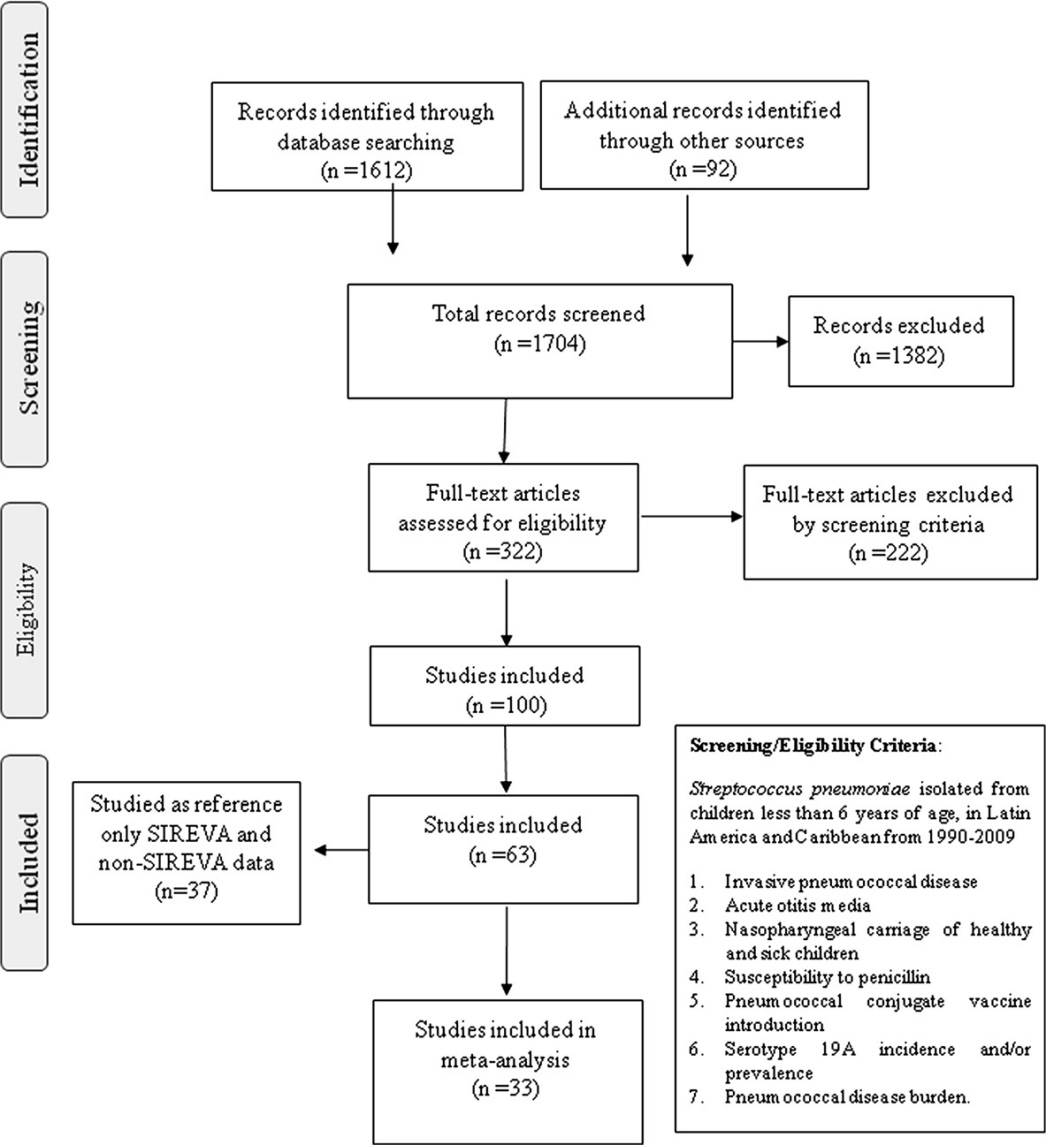
Flow diagram of the literature reviewed.

Our searches retrieved a total of 1704 references. After reviewing the titles and abstracts, a total of 322 full texts were reviewed, 222 of which were excluded. The final number of publications included was 100 and data were extracted from 63; the remaining 37 were referenced (Figure 1). The characteristics of the 63 studies reviewed and of those that were referenced are described in Additional file 2.

The 63 references were divided into three categories: studies with information on invasive serotypes (n = 14), non-invasive isolates with individual information (n = 27) and burden of disease or disease incidence studies (n = 26). Four references provided information on more than one of these categories.

### *Spn *19A in IPD

Most of the IPD isolates identified came from the SIREVA project (19084 of 19733; 96.7%) [23,26]. Of these 19733 isolates, 753 (3.8%) were Spn19A. There was no statistically significant difference in the prevalence of Spn19A isolates between SIREVA (3.8%) and non-SIREVA (3.1%) data (p = 0.3) (Tables 1 and 2). 

**Table 1:** ***Streptococcus pneumoniae *serotype 19A, number of invasive isolates by country, SIREVA data, 1993-2009**

**Country**	**Year of isolation**	**Serotype 19A**		**Total**
		** n**	**%**	**n**
Argentina	1993–2009	100	3.7	2692
Bolivia	2000–2009	5	3.1	160
Brazil	1993–2009	153	3.6	4262
CAREC^a^	2000–2009	2	1.4	148
Chile	1993–2009	161	4.5	3617
Colombia	1993–2009	28	1.5	1819
Costa Rica	2000–2009	3	4.8	63
Cuba	2000–2009	31	3.4	899
DR^b^	2000–2009	14	2.6	544
Ecuador	2000–2009	5	2.3	215
El Salvador	2000–2009	5	9.6	52
Guatemala	2000–2009	11	9.3	118
Honduras	2000–2009	0	0.0	11
Mexico	1993–2009	91	6.2	1463
Nicaragua	2000–2009	2	4.7	43
Panama	2000–2009	15	7.7	194
Paraguay	2000–2009	17	2.2	784
Peru	2000–2009	8	3.2	249
Uruguay	1993–2009	44	3.6	1210
Venezuela	2000–2009	38	7.0	541
**Total**		**733**	**3.8**	**19084**

^a^ CAREC: Caribbean Epidemiology Centre. ^b^ DR: Dominican Republic

References [23,26].

Number of invasive isolates by country. SIREVA data, 1993-2009.

**Table 2:** ***Streptococcus pneumoniae *serotype 19A, number of invasive isolates by country, non SIREVA data, 1989–2008**

**Country [Reference]**	**Year of isolation**	**Serotype 19A**		**Total**
		**n**	**%**	**n**
Argentina [27]^a,b^	1999–2002	0	0.0	153^c^
Brazil [28]^b^	1989–1993	2	4.1	49
Brazil [29]^a^	1995–1999	2	1.4	145
Chile [30]^b^	1994–2004	3	2.3	128
Chile [31]^b^	1995–1997	1	3.3	30
Mexico [32]^b^	1992–1993	11	9.2	120
Colombia [33]^b^	2008	1	4.2	20
**Total**		**20**	**3.1**	**649**

^a^Meningitis.

^b^Unspecified invasive disease.

^c^a = 7, b = 146.

When considering countries with more than 500 isolates collected over a period of 17 years (1993–2009) or 10 years (2000–2009) the total number of invasive isolates was 17831; 677 (3.9%) of these were Spn19A, ranging from 1.5% in Colombia to 7.0% in Venezuela (Table 1).

Analysis of the prevalence of invasive serotypes data from SIREVA (1993–2009) and non SIREVA data (1989–2008) revealed that Spn19A ranked 9^th^ in both sets of data (3.8% SIREVA and 3.1% non-SIREVA (See Additional file 3a)). In an analysis of the SIREVA data in 20 countries, Spn19A ranked 9^th^ (3.6%) for the period 2000–2005, and 8^th^ (4.6%) for the period 2006–2009 (See Additional files 3b and c).

**Figure 2:**
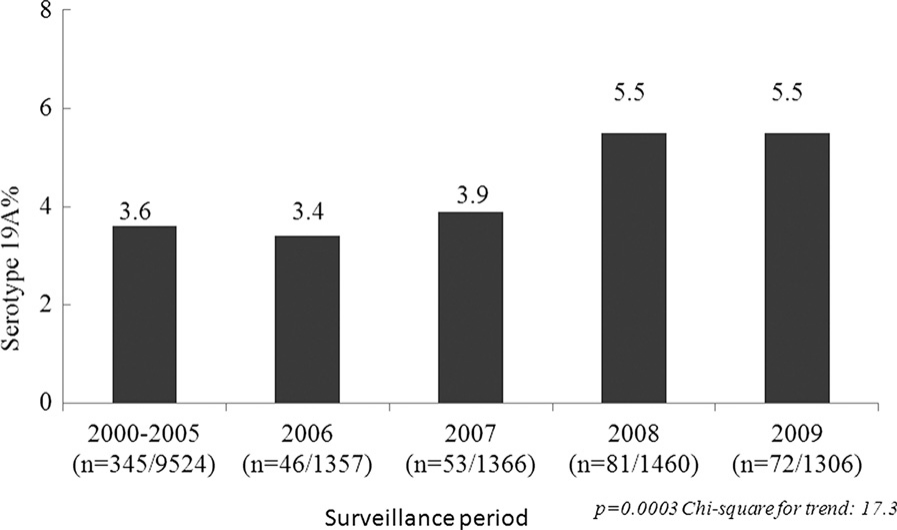
***Streptococcus pneumoniae *serotype 19A distribution of invasive isolates SIREVA data, 2000–2005, 2006, 2007, 2008, 2009.**

The prevalence of Spn19A in the region, by country and by time period is shown in Table 3. Spn19A accounted for 3.3% (CI95%: 1.8–4.9) of isolates between 1993 and 1999, 3.6% (CI95%: 2.3–4.9) between 2000 and 2005 and 4.6% (CI95%: 3.4–5.8) between 2006 and 2009 (*χ*2 10.8, p < 0.001). The prevalence of serotypes recorded from SIREVA data collected during the three time periods presented by vaccine type (VT) (PCV7, PCV10, PCV13) and non vaccine type (NVT), as well as Spn19A, prevalence by region (SIREVA) (Average cases by study period (2000–2005 vs. 2006 – 2009) is shown in Additional file 4. Analysis by SIREVA reports are presented in Figure 2.

**Table 3:** ***Streptococcus pneumoniae *serotype 19A prevalence in 20 countries, SIREVA data, 1993–2009**

**Country**	**Periods analyzed [Reference]**								
	**1993–1999[**[26]**]**			**2000–2005[**[23,34]**]**			**2006–2009[**[23]**]**		
	**19A**		**Total**	**19A**		**Total**	**19A**		**Total**
	**n**	**%**		**n**	**%**		**n**	**%**	
Argentina^a^	30	3.0	1006	33	3.5	936	37	4.9	750
Bolivia	-	-	-	3	2.8	108	2	3.8	52
Brazil	42	3.5	1203	71	3.6	1963	40	3.6	1096
CAREC^b^	-	-	-	1	0.9	110	1	2.6	38
Chile	19	4.1	461	81	4.3	1894	61	4.8	1262
Colombia^a^	4	0.6	623	6	0.9	647	18	3.3	549
Costa Rica	-	-	-	0	0.0	3	3	5.0	60
Cuba	-	-	-	28	3.3	842	3	5.3	57
DR^c^	-	-	-	8	2.1	379	6	3.6	165
Ecuador	-	-	-	0	0.0	55	5	3.1	160
El Salvador	-	-	-	3	13.0	23	2	6.9	29
Guatemala	-	-	-	4	4.4	91	7	25.9	27
Honduras	-	-	-	0	0.0	3	0	0.0	8
Mexico	28	6.6	426	42	5.8	728	21	6.8	309
Nicaragua	-	-	-	1	2.6	39	1	25.0	4
Panama	-	-	-	7	6.9	101	8	8.6	93
Paraguay	-	-	-	12	2.5	477	5	1.6	307
Peru	-	-	-	3	2.1	143	5	4.7	106
Uruguay	13	3.7	352	18	3.1	575	13	4.6	283
Venezuela	-	-	-	24	5.9	407	14	10.4	134
**Total**	**136**	**3.3**	**4071**	**345**	**3.6**	**9524**	**252**	**4.6**	**5489**

^a^ Significant differences between 1993–1999 and 2006–2009 (Argentina p = 0.03, Colombia p < 0.001).

^b^ Caribbean Epidemiology Centre.

^c^ Dominican Republic.

- No data available.

Prevalence in 20 countries. SIREVA data, 1993–2009.

We did not observe any statistical significant differences in the prevalence of Spn19A isolates reported between age groups (4.9% for <2 years vs. 5% for 2–5 years; p = 0.8), for the period 2007–2009 (See Additional file 5).

Spn19A was reported less frequently in meningitis cases (3.0%) than in non-meningitis (4.6%; p < 0.01), with the exception of Paraguay (2.8%vs.2.0%). Differences were statistically significant only for Brazil (3.0% vs. 4.8%; p = 0.009) (See Additional file 6a).

SIREVA data from the period 2006–2007 [23] showed that pneumonia accounted for 59.4% of non-meningitis cases (1069/1801) and 56% of all Spn19A (42/75). Overall, Spn19A accounted for 3.9% of the pneumonia isolates in LAC (See Additional file 6b). Data from 2000–2009, showed that Spn19A was the 10^th^ and 6^th^ most frequently reported serotype causing meningitis and non meningitis, respectively (See Additional files 6c and d).

In non SIREVA data from studies in Argentina [27] and Brazil [29], Spn19A accounted for 1.3% (2/152) of meningitis and none of the 146 non-meningitis.

### *Spn *19A in AOM

Data showing the frequency of serotype 19A amongst isolates in cases of acute otitis media are presented in Table 4. Despite representing more than 70% of the whole sample, only 0.6% of Costa Rican isolates were Spn19A. Data grouped by VT (13, 10 and 7 valent vaccines) and NVT are also shown (See Additional file 7a). Overall, Spn19A accounted for 2.4% (11/460) of isolates, ranking the 9^th^ amongst the most frequent serotype for AOM.

**Table 4:** ***Streptococcus pneumoniae *serotype 19A isolates from acute otitis media and nasopharyngeal - healthy and sick children**

**Country [Reference]**	**Year of isolate**	**Serotype 19A**		**Total**
		**n**	**%**	**n**
**Acute Otitis Media**				
Brazil [35]	1990–1995	6	12.5	48
Colombia [36]	2008–2009	1	2.8	36
Costa Rica [37]	1992–2007	2	0.6	346
Mexico [38]	1995–2000	5	16.7	30
**Total**		**11**	**2.4**	**460**
**Nasopharyngeal isolates - Healthy children**				
Argentina [39]	2000–2007	27	3.7	728
Brazil [40]	1998	1	0.7	135
Brazil [41]	2000	10	4.5	222
Brazil [42]	2000–2001	1	3.0	33
Brazil [43]	2005	21	6.3	332
Chile [44]	1994–1999	0	0.0	68
Chile [45]	1994–1995	5	10.0	50
Chile [46]	2001–2003	16	2.2	714
Colombia [47]	2009	6	3.5	170
Mexico [48]	1994	4	6.2	65
Mexico [38]	1997–2000	6	3.5	173
Mexico [49]	2002	50	6.0	829
Mexico [50]	2002–2003	0	0.0	122
Mexico [51]	2006	19	10.8	176
Panama [52]	2008	2	1.2	163
Peru [53]	2000	3	2.1	146
Uruguay [54]	1993–1995	3	4.1	74
Venezuela [55]	2004–2005	5	4.1	122
Venezuela [56]	2008	1	1.7	58
**Total**		**180**	**4.1**	**4380**
**Nasopharyngeal isolates - Sick children**				
Brazil [57]	1997	3	1.9	162
Brazil [58]	2002–2003	1	8.3	12
Brazil [59]	2009	7	9.9	71
Mexico [48]	1994	5	7.2	69
Uruguay [54]	1993–1995	0	0.0	21
Venezuela [60]	2000	0	0.0	27
**Total**		**16**	**4.4**	**362**

### *Spn *19A in nasopharyngeal carriage

Spn19A data from isolates in healthy children are shown in Table 4; 20 serotypes were identified for 74.7% of nasopharyngeal isolates, of which serotype 19F was the most frequently reported (13.6 and Spn19A the 6^th^ (4.1%) (See Additional file 7b).

The distribution of Spn19A in healthy children (carriage) was similar from that seen for the smaller sample of isolates collected from sick children (Table 4), where Spn19A was the 5^th^ most frequently reported serotype (4.4% of total isolates) (See Additional file 7c).

### *S. pneumoniae* susceptibility to penicillin. Serotype 19A and other serotypes (2007–2009)

A total of 53.7% (22/41) of Spn19A isolates collected from meningitis cases were reported to be resistant. For non-meningitis isolates 10.8% of Spn19A isolates (17/158) showed an intermediate level of resistance and 3.2% (5/158), high resistance (Table 5) [61]. 

**Table 5:** ***Streptococcus pneumoniae *serotype 19A, penicillin resistance, SIREVA data 2007–2009**

**Years[**[23]**]**	***Streptococcus pneumoniae *19A**							
	**Meningitis**			**Non-meningitis**				
	**PNSP resistant**^**a**^		**Total**	**PNSP**				**Total**
				** Intermediate**^**b**^		** High**^**b**^		
	**n**	**%**		**n**	**%**	**n**	**%**	
2007	8	57.1	14	2^**c**^	5.1	0	0.0	39
2008	9	64.3	14	6 ^d^	9.4	3 ^d^	4.7	64
2009	5	38.5	13	9 ^e^	16.4	2 ^e^	3.6	55
**Total**	**22**	**53.7**	**41**	**17**	**10.8**	**5**	**3.2**	**158**

^a^CLSI 2010, Penicillin-Nonsusceptible *S. pneumoniae* (PNSP) resistant ≥ 0.125 μg/ml [61].

^b^CLSI 2010, PNSP intermediate resistance (IR) = 4.0 μg/ml, PNSP high resistance (HR) ≥ 8.0 μg [61].

^c^Brazil (1) MIC 4.0 μg/ml (IR), Venezuela (1) MIC 4.0 μg/ml (IR).

^d^Brazil (2), MIC 4.0 μg/ml (IR); Venezuela (2), MIC 4.0 μg/ml (IR); Chile(1), MIC 4.0 μg/ml (IR); Mexico (1), MIC 4.0 μg/ml (IR); Peru (2), MIC ≥8.0 μg/ml (HR); Colombia (1), MIC ≥8.0 (HR).

^e^Chile (2), MIC 4.0 μg/ml (IR); Colombia (2), MIC 4.0 μg/ml (IR); Cuba (1), MIC 4.0 μg/ml (IR); Dominican Republic (1), MIC 4.0 μg/ml (IR); Mexico (5) 3 MIC 4.0 μg/ml (IR) and 2 MIC ≥8.0 μg/ml (HR).

An analysis of other serotypes showed that among 453 resistant meningeal isolates, Spn19A was the 5^th^ most frequently reported serotype (4.9%) and that, for 248 resistant non-meningitis isolates, it was 3^rd^ (8.3%) (See Additional file 8a). These data are presented by country in Additional file 8b.

### *Spn *19A meta-analysis

**Figure 3:**
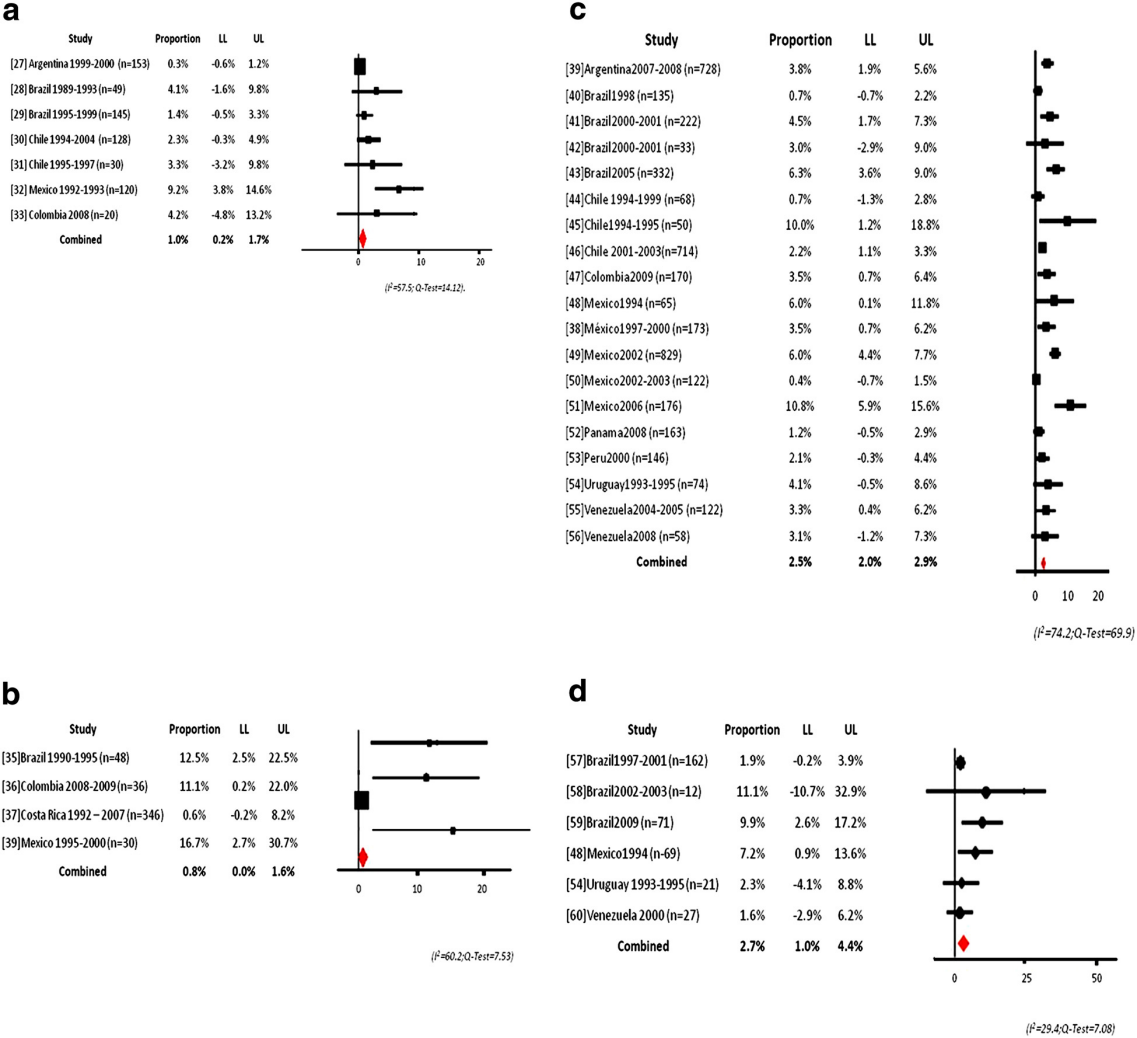
**a Serotype 19A meta-analysis of invasive isolates. b** Serotype 19A meta-analysis of AOM isolates. **c** Serotype 19A meta-analysis healthy nasopharyngeal carriers. **d** Serotype 19A meta-analysis sick carriers.

For invasive disease, our analysis of the data shows 1% prevalence overall for this serotype, ranging from 0.03% in Argentina to 9.2% in Mexico [27-33] (Figure 3a). 

The prevalence of Spn19A in AOM was 0.8%, ranging from 0.6% in Costa Rica to 16.7% in Mexico [35-37,39] (Figure 3b).

The overall prevalence of Spn19A in healthy carriers, for 9 different countries, was 2.5%, ranging from 0.4% for studies conducted in Mexico between 2002 and 2003 period to 10.8% for studies conducted in Mexico in 2006, respectively [38-56] (Figure 3c). For sick children, the overall prevalence for 4 countries was 2.7%, ranging from 1.6% in Venezuela to 11.1% in Brazil [48,54,57-60] (Figure 3d).

### *Spn *19A burden of disease

Our literature search for publications on the burden of disease caused by serotype 19A identified 26 papers; Table 6 summarizes incidence rates reported. Incidences by country are presented in Additional file 9.

**Table 6:** **Estimates of the incidence rate of different pneumococcal diseases in Latin America and the Caribbean and *S. pneumoniae*19A specific incidence**

	**Range expressed in cases per 100,000/year**			
**Condition**	**Age group (years)**			
	** <1**	** <2**	**<3**	** <5**
Invasive pneumococcal disease IPD	61 (58–63)^a^	61 (52–71)		32 (32–33)
*S. pneumoniae* 19A specific incidence^b^	2.3 (2.2–2.9)	2.8 (2.4–3.3)		
Pneumococcal meningitis	19	12		11 (9–15)
*S. pneumoniae* 19A specific incidence^c^	0.6	0.4		0.3 (0.27–0.45)
Pneumococcal pneumonia		51 (47–55)		34
*S. pneumoniae* 19A specific incidence^d^		1.98 (1.8–2.1)		1.3
Pneumococcal sepsis		12		
*S. pneumoniae* 19A specific incidence^e^		0.6		
Pneumococcal bacteremia			2	
*S. pneumoniae* 19A specific incidence^4^			0.2	

(Adapted from reference [62]).

^a^ (25th–75th percentile, when reported).

^b^*S. pneumoniae* 19A distribution reported in SIREVA 1993–2009 = 3.8%.

^c^*S. pneumoniae* 19A distribution reported in SIREVA 2000–2009 = 3.0%.

^d^*S. pneumoniae* 19A distribution reported in SIREVA 2006–2007 = 3.9%.

^e^*S. pneumoniae* 19A distribution reported in SIREVA 2000–2009 = 4.6%.

Lagos *et al.*[46] monitored IPD related hospitalizations in Chile between 1994 and 2007. Among the serotypes identified, “other” or “non-vaccine serotypes within vaccine serogroups” (which included Spn19A) were reported for 72 patients with invasive clinical syndromes. For these patients, the case fatality rate was 0%. The annual incidence of IPD among children 0–59 months of age caused by Spn19A was 1 per 100,000.

In Córdoba, Argentina, Tregnaghi *et al.*[27] found a highest incidence of IPD (206/100,000 children <2 years old), attributed to ambulatory patients. No Spn19A isolates were isolated.

#### Pneumococcal vaccine potential impact for invasive disease

With the SIREVA data for IPD the estimated percentage was 63% for PCV7, 79.3% for PCV10, and 85.5% for PCV13. We carried out an analysis to establish the potential benefit of adding Spn19A, 1 and 5. This showed that addition of serotypes 1 and 5 increase impact by 13.2%, whilst addition of 19A increases vaccine impact by 4% (see Additional file 10).

## Discussion

This systematic review of Spn19A data in children under 6 years old, from studies conducted in LAC over a period of 20 years shows that Spn19A remains a less common agent of IPD than other serotypes (3.8%), ranking 9^th^ in the twenty most prevalent serotypes [23]. The percentage of isolates accounted by Spn19A differed between countries, being the 10^th^ most frequently reported from Colombia, the 6^th^ from Mexico and 4th from Venezuela (Additional file 3a and Additional file 11). This information provides a complete overview of the role of Spn19A for pneumococcal disease facilitating the decision process for those countries considering to introduce PCV, but also will allow evaluation of potential variations in the prevalence of Spn19A and other serotypes, as reported previously in studies following introduction of PCV7 [14-19,23].

Our analysis of the literature identified the serotypes accounting for 85.4% of IPD in the region, serotype 14 being the most common (28.7%). However, the percentage of isolates accounted for by each of these serotypes varied from country to country, in agreement with JohnsonÂ´s observation in her recent global serotype paper [4].

The scope of our search strategy allowed us to retrieve comprehensive lists of peer-reviewed publications. Two of our authors being members of the SIREVA team, we were able to identify the vast majority of relevant publications in non-indexed journals and obtain personal communications with SIREVA coordinators [23]. Additionally, information retrieved from over a 20 year period evaluated secular trends and the periodicity of serotypes described in the literature [1-4].

A strength of our analysis is that the percentage of IPD Spn19A isolates reported in the non SIREVA data that we reviewed (7 reports, 1990–2008) was not significantly different from that for the SIREVA data (3.8%).

Regarding time period of Spn19A prevalence, a significant increase, from 3.3% to 4.6%, was noted only in Argentina and Colombia between 1994–1999 and 2006–2009 before any universal vaccine intervention could have had an impact. However, Spn19A stability was observed in Brazil, Chile, Dominican Republic and Mexico. Similar increases in the percentage of isolates accounted for Spn19A, even prior to the introduction of PCV7, have been reported in Europe [63], South Korea [64], Southern Israel [65] and Taiwan [66], likely reflecting selection pressure from antibiotic use.

On the other hand, in the USA the observed increasing prevalence of PRSP and MDR Spn19A has been suggested to be due to a rapid expansion of the Spn19A clonal complex CC320, to more than one new clone introduced or to successful clones associated with other serotypes that have undergone a recombinational switch to Spn19A [20,21].

In the LAC region only one study, describing PFGE patterns of Spn19A isolates, and conducted in Colombia [67], reported Spn19A MDR isolates in IPD; two were found related to the clone Colombia^23F^-ST338, one to the clone Spain^23F^-ST81, and 6 were not related to the clones studied. A possible explanation of these findings may be that a successful clone, such as Spain^23F^, underwent a recombinational switch to Spn19A.

No differences could be established between age groups for the prevalence of Spn19A as an IPD agent. In contrast, serotypes 1 and 5 were more frequent in children 2–5 years old and serotypes 6B and 14 were more frequent in <2 year olds than in the other age groups in the LAC region (See Additional file 5).

Our analysis suggests that Spn19A causing IPD in LAC is more frequently an agent of non-meningitis disease (4.5% of cases), in particular pneumonia than of meningitis (2.9%) (See Additional file 6a).

PNSP in invasive Spn19A isolates has been reported in LAC since 1993 [26]. A study conducted in 2010, using the new CLSI breakpoints for penicillin [61], showed that resistant Spn19A isolates are circulating in the region, more frequently as agents of meningitis (MIC ≥ 0.125 μg/ml) than for of non-meningitis (MIC ≥ 4.0 μg/ml). However, the finding of a prevalence of 3.2% for Spn19A with MIC ≥ 8.0 μg/ml among non-meningitis cases, recovered in Mexico, Colombia and Venezuela is of great concern, as it follows reports of 7.7% of cases being attributed to serotype 19F. Molecular surveillance data will reveal their role as agents of pneumococcal disease [20,21].

Despite the fact that *S. pneumoniae* causes 30–60% of AOM cases worldwide [68], only three papers and one abstract were found and analyzed; overall, 2.4% of these were attributed to Spn19A. As AOM continues to be an important childhood infection and given that the etiology might change from VT to non-PCV7 strains once pneumococcal vaccines are widely implemented [69], it is important to conduct AOM etiology studies in the region. *S. pneumoniae* may be subject to serotype replacement phenomena and attention to antibiotic resistant NVT otopathogens as well as non typable *Haemophilus influenzae* is required [70].

Nasopharyngeal carriage has been confirmed with greater values reported for children less than 5 years old. From the papers analyzed, Spn19A ranked the 6^th^ most frequently reported serotype for healthy children (4.1%), jointly with Non-Typable. There were a high number of serotypes with ability for colonizing the nasopharynx, with serotype 19 F the most frequently identified (See Additional file 7b).

Nasopharyngeal serotypes described in Latin America from 1994 to 2008 are very similar to those described by Huang in 2001 (pre vaccine data) for generally healthy children in 16 Massachusetts communities. Spn19A represented 4.2% of 143 isolates; PNSP was described for 77% of the NVT, in particular for serotypes 6A, 19A and 9A [12].

Studies conducted since the introduction of PCV7 vaccination have shown decreases in colonization with pneumococcal VT shortly after immunization as well as longer-term changes in colonization patterns. Huang [12] reported a decrease in the carriage of VT from 36% to 3% seven years after mass introduction of PCV7, whereas NVT carriage increased from 15% to 29%. The common colonizing serotypes in 2007 included 19A (16%) (Baseline data 6.0%), 6A (12%), 15B/C (11%), 35B (9%) and 11A (8%), a clear reflection of the replacement phenomenon. Additionally, the more frequent colonizing serotypes have greater resistance to penicillin. Nasopharyngeal surveillance appears to be a reliable system for measuring vaccination impact in terms of a decrease in VT types and will help to elucidate the emergence of NVT following PCV introduction.

Incidence rates reported by Lagos [46] for IPD caused by Spn19A ranged from 0.4 to 2.2 cases x 100,000 between 1994 and 2007, suggesting a seasonal pattern for this serotype. Similar variations have been shown for other serotypes in the LAC region, such as 1 and 5 [34] and may explain changes during time periods in the SIREVA data presented in this review. This should be considered when interpreting data post introduction of pneumococcal vaccines in this region. In contrast, the incidence of other serotypes such as 14 has shown small variations [26,34].

PCVs have been introduced recently in several countries in LAC, but currently, there are no data published about their impact in reducing IPD. Consequently, little is known about the replacement phenomenon with Spn19A, which has been well described previously [22]. Available data provide only an estimation of hypothetical impact (supplement 10). The same calculation for the recent SIREVA data [4] showed a major impact of PCV10 and PCV13 vaccination, in particular related to the inclusion of serotypes 1 and 5. In fact, after 2009, countries in the region have incorporated different PCV into their expanded program of immunization following individual assessment for their epidemiology (PCV7/13: Costa Rica, Uruguay, Mexico and PCV-10: Brazil, Colombia, Ecuador and Chile). Following the results of this review, indicating the low prevalence of Spn19A in most of the countries, it is necessary to report any subsequent change in the distribution of this serotype in those countries who have introduced one of the available PCV. Particularly, trying to explain any increase or decrease in the Spn19A prevalence comparing the statistics prior to universal vaccination and possible factors that could explain this, such as vaccine coverage, antibiotic use and immune response based on the vaccine formulation.

The results of our systematic review have a number of limitations. The source of primary data, either from SIREVA or from independent research teams, could introduce selection bias, potentially promoting the selection of more severe forms of the disease. However, it is important to highlight that more severe disease will have the largest impact from a burden of disease or a public health perspective. Information on disease severity caused by Spn19A in this region was limited; this is also the case for data collected for other serotypes, given that similar surveillance activities are employed in the different countries. As this limitation is not restricted to a specific serotype, it should not bias our conclusions. It was not possible to analyze temporal changes in serotype frequency, except from a very broad perspective. The small amount of data available on burden of disease and on the possible effects of mass vaccination highlights the need for more research in this area.

Incidence of IPD in this region ranges from 3.0 to 206.8 cases per 100,000. Overall 9 serotypes are responsible for 80% of IPD and 30% are due to serotype 14; Spn19A remains relatively uncommon as an agent of IPD, with the exception of Mexico and Venezuela. Data on noninvasive disease and nasopharyngeal carriage, although not as robust, show the same low prevalence.

## Conclusions

As several countries in the region implemented PCV in their routine schedules starting in 2006, regional data on vaccination impact on IPD, non invasive and nasopharyngeal carriage by VT and herd effect should soon be available. In the near future we expect that data on VT and NVT, supported by a solid surveillance system, will be available, which will support public health decisions on the introduction of PCV.

## Competing interests

Elizabeth Castañeda, Clara Inés Agudelo, Diego Rosselli and Claudia Calderón no conflicts to declare.

Rodrigo De Antonio, Eduardo Ortega-Barria and Romulo E Colindres are employees of GlaxoSmithKline Biologicals.

Eduardo Ortega-Barria and Rómulo E Colindres have stock ownership; Rodrigo DeAntonio has stock options.

## Authors’ contributions

EC contributed to systematic review conception and design, data analysis, interpretation of data, elaboration, review and comments on all drafts of this paper and gave final approval to submit for publication. CIA contributed to systematic review conception and design, data analysis, interpretation of data, elaboration, review and comments on all drafts of this paper and gave final approval to submit for publication. RDA contributed to systematic review conception and design, data analysis, interpretation of data, review and comments on all drafts of this paper and gave final approval to submit for publication. DR contributed to systematic review conception and design, data analysis, interpretation of data, review and comments on all drafts of this paper and gave final approval to submit for publication. CC contributed to systematic review conception and design, data analysis, interpretation of data, review and comments on all drafts of this paper and gave final approval to submit for publication. EO-B contributed to systematic review conception and design, interpretation of data, review and comments on all drafts of this paper and gave final approval to submit for publication. REC contributed to systematic review conception and design, interpretation of data, review and comments on all drafts of this paper and gave final approval to submit for publication. All authors read and approved the final manuscript.

## Pre-publication history

The pre-publication history for this paper can be accessed here:

http://www.biomedcentral.com/1471-2334/12/124/prepub

## Supplementary Material

Additional file 1 **a- *Streptococcus pneumoniae *serotype 19A, search strategies.** b - *Streptococcus pneumoniae* definitions. Click here for file

Additional file 2 **Characteristics of the 63 studies included in the analysis, SIREVA and non SIREVA [**[2]**,**[27]**-**[60]**,**[62]**,**[71]**-**[135]**].** Click here for file

Additional file 3 **a- *Streptococcus pneumoniae.*** Number of invasive isolates by serotype and country presented by vaccine type (VT) (PCV7, PCV10, PCV13) and non vaccine type (NVT). SIREVA, 1993–2009 and non SIREVA, 1989–2008. b - *Streptococcus pneumoniae*. Serotypes invasive isolates, < 6 years old, SIREVA, 20 countries*, 2000–2005. c - *Streptococcus pneumoniae*. Serotypes invasive isolates, < 5 years old, SIREVA, 20 countries*, 2006–2009 [27-33,75,82,85,86,89,97]. Click here for file

Additional file 4 ***Streptococcus pneumoniae, *serotype prevalence in three time periods of SIREVA results presented by vaccine type (VT) (PCV7, PCV10, PCV13) and non vaccine type (NVT).**[75,82,85,86,89,97]. Click here for file

Additional file 5 ***Streptococcus pneumoniae.*** Serotype distribution for invasive isolates by age group <2 years and 2–5 years. SIREVA data, 2007–2009 [86,89,97]. Click here for file

Additional file 6 **a- *Streptococcus pneumoniae *serotype 19A.** Meningitis, non-meningitis in ten countries. SIREVA data, 2000–2009. b - *Streptococcus pneumoniae* serotype 19A. Non-meningitis and pneumoniae cases in 20 countries. SIREVA data, 2006*–*2007. c - *Streptococcus pneumoniae*. Meningitis isolates by country and serotype presented by vaccine type (VT) (PCV7, PCV10, PCV13) and non vaccine type (NVT). SIREVA data, 2000*–*2009. d - *Streptococcus pneumoniae*. Non-meningitis isolates by country and serotype presented by vaccine type (VT) (PCV7, PCV10, PCV13) and non vaccine type (NVT). SIREVA data, 2000–2009 [34,82,85,86,89,97]. Click here for file

Additional file 7 **a- *Streptococcus pneumoniae *.** Number of acute otitis media isolates presented by vaccine type (VT) (PCV7, PCV10, PCV13) and non vaccine type (NVT). b - *Streptococcus pneumoniae*. Number of nasopharyngeal isolates in healthy children presented by vaccine type (VT) (PCV7, PCV10, PCV13) and non vaccine type (NVT). c - *Streptococcus pneumoniae*. Number of nasopharyngeal isolates in sick children presented by vaccine type (VT) (PCV7, PCV10, PCV13) and non vaccine type (NVT) [35-60,76]. Click here for file

Additional file 8 **a- *Streptococcus pneumoniae *.** Meningitis and non-meningitis isolates, penicillin resistance by serotype. SIREVA data 2007–2009. b - *Streptococcus pneumoniae* serotype 19A. Meningitis isolates, penicillin resistance. Argentina, Brazil, Colombia, Mexico and Venezuela. 2007–2009 [86,89,97]. Click here for file

Additional file 9 **Pneumococcal diseases in Latin America and the Caribbean incidences by country [**[27]**,**[29]**,**[33]**,**[46]**,**[77]**-**[79]**,**[83]**,**[134]**,**[135]**].** Click here for file

Additional file 10 **Pneumococcal vaccines.** Hypothetical vaccine impact, population <6 years [27-30,32-36,40-42,46,49,50,55,56,58,74,75,82,85-90,97,108,112,113,117,119,123,129]. Click here for file

Additional file 11 ***Streptococcus pneumoniae *serotype 19A prevalence in the region.** SIREVA data 2000–2009. Click here for file
