# Supplementary material for: Streptococcus pneumoniae serotype 19A in Latin America and the Caribbean: a systematic review and meta-analysis, 1990–2010
Source: BMC Infect Dis. 2012 May 28;12:124. doi: 10.1186/1471-2334-12-124 (PMC3475047; doi:10.1186/1471-2334-12-124)
Supplement: Additional file 1 — a- Streptococcus pneumoniae serotype 19A, search strategies. b - Streptococcus pneumoniae definitions. [file 1471-2334-12-124-S1.doc]

**Supplement 1**

**Supplement 1a. *Streptococcus pneumoniae* serotype 19A, s**earch strategies

| ***Search strategy #1***  "Streptococcus pneumoniae"[Mesh] AND "Latin America"[Mesh] |
| --- |
| ***Search strategy #2***  "sireva [All Fields] |
| ***Search strategy #3***  "Streptococcus pneumoniae"[Mesh] AND (“Caribbean Region” OR “West Indies” OR “Central America” OR “Belize” OR “Costa Rica” OR “El Salvador” OR “Guatemala” OR “Honduras” OR “Nicaragua” OR “Panama” OR “Latin America” OR “Mexico” OR “South America” OR “Argentina” OR “Bolivia” OR “Brazil” OR “Chile” OR “Colombia” OR “Ecuador” OR “Paraguay” OR “Peru” OR “Uruguay” OR “Venezuela” OR “Antigua and Barbuda” OR Bahamas OR Barbados OR Cuba OR Dominica OR “Dominican Republic” OR Grenada OR Guadeloupe OR Haiti OR Jamaica OR Martinique OR “Netherlands Antilles” OR “Saint Kitts and Nevis” OR “Saint Lucia” OR “Saint Vincent and the Grenadines” OR “Trinidad and Tobago”)  Limits Activated: only items with abstracts, Humans, English, Spanish, Portuguese, All Child: 0-18 years, Publication Date from 1985 |
| **Search strategy #4**  “serotype 19A” (99) AND (Caribbean Region” OR “West Indies” OR “Central America” OR “Belize” OR “Costa Rica” OR “El Salvador” OR “Guatemala” OR “Honduras” OR “Nicaragua” OR “Panama” OR “Latin America” OR “Mexico” OR “South America” OR “Argentina” OR “Bolivia” OR “Brazil” OR “Chile” OR “Colombia” OR “Ecuador” OR “Paraguay” OR “Peru” OR “Uruguay” OR “Venezuela” )  NOT #1 NOT #2 NOT #3 |
| **Search strategy #5**  “sp19A” (2) |

**Supplement 1b.** Streptococcus pneumoniae definitions

| ***Invasive pneumococcal disease* *(IPD)*** consists of any of the following possible diagnoses: pneumonia defined using the WHO clinical criteria, with or without radiologic results, meningitis, sepsis, or bacteremia without focus; less frequently reported disease forms were arthritis, peritonitis, sinusitis, abscesses, and cellulitis. Following SIREVA criteria, we divided invasive pneumococcal disease into meningitis or “other”. In some papers results were grouped together in a third category: “unspecified invasive disease”. |
| --- |
| ***For acute otitis media (AOM)*** we identified studies with serotype/serogroup data on *S. pneumoniae* isolates obtained from middle ear fluid of children under five years defined with AOM signs/symptoms (otalgia /irritability, conjunctivitis, fever and bulging, diffused or localized inflamed tympanic membranes or spontaneous otorrhea). Publications with data for recurrent AOM (>3 episodes of otitis media in the previous 6 months or >4 episodes in the previous 12 months) and treatment failures (persistent symptoms after 72h or within 14 days of the last dose of an antibiotic prescribed) were included. |
| ***Nasopharyngeal carriage*** contain studies assessing the prevalence of *S. pneumoniae* serotypes in the nasopharynx of healthy children (attending day-care centers and primary schools) and sick children (hospitalized and/or children with medical condition). |
