# Supplementary material for: Streptococcus pneumoniae serotype 19A in Latin America and the Caribbean: a systematic review and meta-analysis, 1990–2010
Source: BMC Infect Dis. 2012 May 28;12:124. doi: 10.1186/1471-2334-12-124 (PMC3475047; doi:10.1186/1471-2334-12-124)
Supplement: Additional file 2 — Characteristics of the 63 studies included in the analysis, SIREVA and non SIREVA [[2],[27]-[60],[62],[71]-[135]]. [file 1471-2334-12-124-S2.docx]

**Supplement 2**

**Characteristics of the 63 studies included in the analysis, SIREVA and non SIREVA**

| **Reference** | **Study** | **Country (ies)** | **Age group** | **Time period** | **Category^a^** |
| --- | --- | --- | --- | --- | --- |
| **[32]** | Echániz-Aviles G. Capsular types of *Streptococcus pneumoniae* causing disease in children from Mexico city. Pediatr Infect Dis J. 1995; 14:907-909. | Mexico | <5 years | 1992- 1993 | 2 |
| **[28]** | Berezin EN, Carvalho ES, Casagrande S, Brandileone MC, Mimica IM, Farhat CK. *Streptococcus pneumoniae* penicillin-nonsusceptible strains in invasive infections in Sao Paulo, Brazil. Pediatr Infect Dis J. 1996; 15:1051-1053. | Brazil | <2 years | 1989- 1993 | 2 |
| **[45]** | Trucco OA, Prado JV, Inostroza SJ, Cabrera JE, Teran RR, Bravo PI, Castillo DL, Bustos VR. Prevalencia de *Streptococcus pneumoniae* resistente a penicilina en niños que asisten a jardines infantiles en Santiago. Rev Chil Pediatr.1996; 67:195-199. | Chile | 0-4 years | 1994-1995 | 3 |
| **[71]** | Lopez Bravo I, Sepulveda H, Valdes I. Acute respiratory illnesses in the first 18 months of life. Rev Panam Salud Pública. 1997; 1:9-17. | Chile | <18 months | 1991-1992 | 4 |
| **[48]** | Miranda Novales MG, Solórzano Santos F, Guiscafre Gallardo H, Leaños Miranda B, Echániz-Aviles G, Carnalla Barajas MN, Palafox Torres M, Muñoz Hernández O. *Streptococcus pneumoniae*: low frequency of penicillin resistance and high resistance to trimethoprim-sulfamethoxazole in nasopharyngeal isolates from children in a rural area in Mexico. Arch Med Res. 1997; 28:559-563. | Mexico | <5 years | 1994 | 3 |
| **[31]** | Inostroza J, Trucco O, Prado V, Vinet AM, Retamal G, Ossa G, Facklam RR, Sorensen RU. Capsular serotype and antibiotic resistance of *Streptococcus pneumoniae* isolates in two Chilean cities. Clin Diag Lab Immunol. 1998; 5:176-180. | Chile | <2 years | 1995-1996-1997 | 2 |
| **[54]** | Palacio R, Camou T, Russi JC, Hortal M, Picon T, Nin M, Quian J. Frequency, type and associated diseases of bacteria and virus in the oropharynx of children born to human immunodeficiency virus-infected mothers. Braz J Infect Dis. 1998; 2:128-134. | Uruguay | <5 years | 1993-1995 | 3 |
| **[44]** | Gherardi G, Inostroza JS, O'Ryan M, Prado V, Prieto S, Arellano C, Facklam RR, Beal LB. Genotypic survey of recent beta-lactam-resistant pneumococcal nasopharyngeal isolates from asymptomatic children in Chile. J Clin Microbiol. 1999; 37:3725-3730. | Chile | 6-48 years | 1994-1999 | 3 |
| **[72]** | Magnus SA, Hambleton IR, Moosdeen F, Serjeant GR. Recurrent infections in homozygous sickle cell disease. Arch Dis Child. 1999; 80:537-541. | Jamaica | <5 years | 1974-1997 | 4 |
| **[73]** | Gómez E, Peguero M, Sánchez J, Castellanos PL, Feris J, Peña C, Brudzinski-LaClaire L, Levine OS. Population-based surveillance for bacterial meningitis in the Dominican Republic: implications for control by vaccination. Epidemiol Infect. 2000; 125:549-554. | Dominican Republic | <5 years | 1998 | 4 |
| **[74]** | Hortal M, Camou T, Palacio R, Dibarboure H, García A. Ten-year review of invasive pneumococcal diseases in children and adults from Uruguay: Clinical spectrum, serotypes, and antimicrobial resistance. Int J Infect Dis. 2000; 4:91-95. | Uruguay | <5 years | 1987-1997 | 4 |
| **[75]** | Di Fabio JL, Hortal M, Ruvinsky R, Rossi A, Agudelo CI, Castañeda E, Brandileone C, Camou T, Palacio R, Echániz-Aviles G. Evolution of *Streptococcus pneumoniae* serotypes and penicillin susceptibility in Latin America, Sireva-Vigía Group, 1993 to 1999. Ped Infect Dis J. 2001; 20:959-967. | Argentina, Brazil, Chile, Colombia, Mexico, Uruguay | <6 years | 1993-1999 | 1 |
| **[35]** | Sih TM. Acute otitis media in Brazilian children: analysis of microbiology and antimicrobial susceptibility. Ann Otol Rhinol Laryngol. 2001; 110:662-666. | Brazil | 0-5 years | 1990-1995 | 3 |
| **[76]** | Gómez-Barreto D, Rodríguez RS, Calderón Jaimes E, Espinoza LE. Bases fisiopatológicas para la prevención de las infecciones por *Streptococcus pneumoniae*. Boletín Médico del Hospital Infantil de México. 2001; 58:866–878. | Mexico | <4 years | 1995-2000 | 3 |
| **[77]** | Weiss D, Coplan P, Guess H. Epidemiology of bacterial meningitis among children in Brazil, 1997-1998. Rev Saúde Pública. 2001; 35:249-255. | Brazil | < 5 years | 1997-1998 | 4 |
| **[53]** | Cullotta AR, Kalter HD, Delgado J, Gilman RH, Facklam RR, Velapatino B, Coronel J, Cabrera L, Urbina M. Antimicrobial susceptibilities and serotype distribution of *Streptococcus pneumoniae* isolates from a low socioeconomic area in Lima, Peru. Clinical and Diagnostic Lab Immunol. 2002; 9:1328-1331. | Peru | < 39 months | 2000 | 3 |
| **[29]** | Reis JN, Cordeiro SM, Coppola SJ, Salgado K, Carvalho MG, Teixeira LM, Thompson TA, Facklam RR, Reis MG, Ko AI. Population-based survey of antimicrobial susceptibility and serotype distribution of *Streptococcus pneumoniae* from meningitis patients in Salvador, Brazil. J Clin Microbiol. 2002; 40:275-277. | Brazil | <5 years | 1995-1999 | 2 |
| **[38]** | Gómez-Barreto D, Calderón-Jaimes E, Rodríguez RS, Espinosa LE, Viña-Flores L, Jiménez-Rojas V. Carriage of antibiotic-resistant pneumococci in a cohort of a daycare center. Salud Pública Mex. 2002; 44:26-32. | Mexico | <4 years | 1997-1999 | 3 |
| **[78]** | Asturias EJ, Soto M, Menendez R, Ramirez PL, Recinos F, Gordillo R, Holt E, Halsey NA. Meningitis and pneumonia in Guatemalan children: the importance of *Haemophilus influenzae* type b and *Streptococcus pneumoniae*. Rev Panam Salud Pública. 2003; 14:377-384. | Guatemala | <5 years | 1996 -1999 | 4 |
| **[40]** | Lucarevschi BR, Baldacci ER, Bricks LF, Bertoli CJ, Teixeira LM, Mendes CM, Oplustil C. Oropharyngeal carriage of *Streptococcus pneumoniae* by children attending day care centers in Taubate, SP: Correlation between serotypes and the conjugated heptavalent pneumococcal vaccine. J Pediatr (Rio J). 2003; 79:215-220. | Brazil | 8-71 months | 1998 | 3 |
| **[79]** | Ulloa Gutierrez R, Avila-Aguero ML, Herrera ML, Herrera JF, Arguedas A. Invasive pneumococcal disease in Costa Rican children: a seven year survey. Pediatr Infect Dis J. 2003; 22:1069-1074. | Costa Rica | <5 years | 1995-1999 | 4 |
| **[80]** | McGregor D, Barton M, Thomas S, Christie CD. Invasive pneumococcal disease in Jamaican children. Ann Trop Pediatr. 2004; 24:33-40. | Jamaica | <5 years | 1995-1999 | 4 |
| **[58]** | Fonseca PB, Braga JÁ, Machado AM, Brandileone MC, Farhat CK. Colonizacao nasofaringea pelo *Streptococcus pneumoniae* em criancas com doenca falciforme usando penicilina profilatica. J Pediatr (Rio J). 2005; 81:149-154. | Brazil | <6 years | 2002- 2003 | 3 |
| **[50]** | Solórzano-Santos F, Ortiz-Ocampo LA, Miranda-Novales MG, Echániz-Aviles G, Soto-Noguerón A, Guiscafré-Gallardo H. Serotipos prevalentes de *Streptococcus pneumoniae* colonizadores de nasofaringe, en niños del Distrito Federal. Salud Pública Mex. 2005; 47:276-281. | Mexico | 0-5 years | 2002-2003 | 3 |
| **[41]** | Laval CB, de Andrade AL, Pimenta FC, de Andrade JG, de Oliveira RM, Silva SA, de Lima EC, Fabio JL, Casagrande ST, Brandileone MC. Serotypes of carriage and invasive isolates of *Streptococcus pneumoniae* in Brazilian children in the era of pneumococcal vaccines. Clin Microbiol Infect. 2006; 12:50-55. | Brazil | <5 years | 2000-2001 | 3 |
| **[60]** | Quintero B, Araque M. Perfil serológico y antibiotipia de cepas de *Streptococcus pneumoniae* aisladas de portadores nasales pediátricos. Invest Clin. 2006; 47:17-26. | Venezuela | 28 days - 5 years | 2000 | 3 |
| **[27]** | Tregnaghi M, Ceballos A, Ruttimann R, Peeters P, Tregnaghi JP, Ussher J, Rodriguez M, Carvajal L, Yudowski S, Bortolin A, De Lisa I, Oller A, Robledo H. Vigilancia epidemiológica activa de la enfermedad neumocócica en lactantes, en el ámbito ambulatorio y en la internación. Arch Argent Pediatr. 2006; 104:3-9. | Argentina | 2-23 months | 1999-2002 | 2, 4 |
| **[57]** | Berezin EN, Cardenuto MD, Ferreira LL, Otsuka M, Guerra ML, Brandileone MC. Distribution of *Streptococcus pneumoniae* serotypes in nasopharyngeal carriage and in invasive pneumococcal disease in Sao Paulo, Brazil. Pediatr Infect Dis J. 2007; 267:643-645. | Brazil | <5 years | 1997-2001 | 3 |
| **[81]** | Constenla D. Evaluating the costs of pneumococcal disease in selected Latin American countries. Rev Panam Salud Pública. 2007; 22:268-278. | Brazil, Chile and Uruguay | <60 months | 2001 | 4 |
| **[49]** | Espinosa-de Los Monteros LE, Jiménez-Rojas V, Aguilar-Ituarte F, Cashat-Cruz M, Reyes-López A, Rodríguez-Suárez R, Kuri-Morales P, Tapia-Conyer R, Gómez-Barreto D. *Streptococcus pneumoniae* isolates in healthy children attending day-care centers in 12 states in Mexico. Salud Pública Mex. 2007;49:249-255. | Mexico | 2 months to 6 years | 2002 | 3 |
| **[82]** | Grupo de Trabajo de SIREVA II. Informe Regional de SIREVA II: Organización Panamericana de la Salud. Informe Regional de SIREVA II, 2000-2005: datos por país y por grupo de edad sobre las características de los aislamientos *de Streptococcus pneumoniae, Haemophilus influenzae* y *Neisseria meningitidis* en procesos invasivos, **2000-2005.** (Serie Documentos técnicos. Tecnologías esenciales de salud. THS/EV-2007/002). Washington: OPS; 2007. | LAC^b^ | <6  years | 2000-2005 | 1 |
| **[83]** | Hortal M, Estevan M, Iraola I, De Mucio B. A population-based assessment of the disease burden of consolidated pneumonia in hospitalized children under five years of age. Int J Infect Dis. 2007; 11:273-277. | Uruguay | 0-59 months | 2001-2004 | 4 |
| **[30]** | Inostroza J, Illesca V, Reydet P, Vinet AM, Ossa G, Muñoz S, Thompson T, Sorensen RU. Ten-year surveillance of pneumococcal infections in Temuco, Chile: implications for vaccination strategies. Clin Vaccine Immunol. 2007; 14:660-664. | Chile | <5 years | 1994-2004 | 2 |
| **[55]** | Rivera-Olivero IA, Bogaert D, Bello T, del Nogal B, Sluijter M, Hermans PW, de Waard JH. Pneumococcal carriage among indigenous Warao children in Venezuela: serotypes, susceptibility patterns, and molecular epidemiology. Clin Infect Dis. 2007; 45:1427-1434. | Venezuela | 0-72 months | 2004-2005 | 3 |
| **[84]** | Constenla DO. Economic impact of pneumococcal conjugate vaccination in Brazil, Chile, and Uruguay. Rev Panam Salud Pública. 2008; 24:101-112. | Brazil, Chile and Uruguay | <5 years | 2007 | 4 |
| **[85]** | Grupo de Trabajo de SIREVA II. Informe Regional de SIREVA II: Organización Panamericana de la Salud. Informe Regional de SIREVA II, 2006: datos por país y por grupo de edad sobre las características de los aislamientos de *Streptococcus pneumoniae*, *Haemophilus influenzae* y *Neisseria meningitidis* en procesos invasivos, 2006. (Serie Documentos técnicos. Tecnologías esenciales de salud. THS/EV-2008/001). Washington: OPS; 2008. | LAC^b^ | <5 years | 2006 | 1 |
| **[86]** | Grupo de Trabajo de SIREVA II. Informe Regional de SIREVA II**:** Organización Panamericana de la Salud. Informe Regional de SIREVA II, 2007: datos por país y por grupo de edad sobre las características de los aislamientos de *Streptococcus pneumoniae, Haemophilus influenzae* y *Neisseria meningitidis* en procesos invasivos, 2007. (Serie Documentos técnicos. Tecnologías esenciales de salud. THS/EV-2008/003). Washington: OPS; 2008. | LAC^b^ | <5 years | 2007 | 1 |
| **[46]** | Lagos R, Muñoz A, San Martin O, Maldonado A, Hormazabal JC, Blackwelder WC, Levine MM. Age- and serotype-specific pediatric invasive pneumococcal disease: Insights from systematic surveillance in Santiago, Chile, 1994 - 2007. J Infect Dis. 2008; 198:1809-1817. | Chile | 0-23 months | 2001-2003 | 3,4 |
| **[87]** | Mattei SM, Falleiros-Carvalho LH, Cavalcante NJF. Invasive pneumococcal disease in HIV seropositive children and adolescents. J Pediatr (Rio J). 2008; 84:276-280 | Brazil, Chile, Uruguay | <5 years | 1993-2000 | 4 |
| **[42]** | Reis JN, Palma T, Ribeiro GS, Pinheiro RM, Ribeiro CT, Cordeiro SM, da Silva Filho HP, Moschioni M, Thompson TA, Spratt B, Riley LW, Barocchi MA, Reis MG, Ko AI. Transmission of *Streptococcus pneumoniae* in an urban slum community. J Infect. 2008; 57:204-213. | Brazil | <5 years | 2000-2001 | 3 |
| **[51]** | Reyna J, Limon AE. High prevalence of serotype 11B of *Streptococcus pneumoniae* isolated in the nasopharynx of Mexican children. Arch Med Res. 2008; 39:629-630. | Mexico | ≤5 years | 2006 | 3 |
| **[33]** | Benavides JA, Ovalle OO, Salvador GR, Gray S, Isaacman D, Rodgers GL. Population-based surveillance for invasive pneumococcal disease and pneumonia in infants and young children in Bogotá, Colombia. Presented at: 6th International Symposium on Pneumococci and Pneumococcal Diseases (ISPPD-7). Edited by. Islandia: Reykjavik; 2008. Available at Vaccine. 2012 Apr 4. [Epub ahead of print] | Colombia | <2 years | 2006 | 2,4 |
| **[37]** | Abdelnour A, Soley C, Guevara S, Porat N, Dagan R, Arguedas A. *Streptococcus pneumoniae* serotype 3 among Costa Rican children with otitis media: Clinical, epidemiological characteristics and antimicrobial resistance patterns. BMC Pediatrics. 2009; 9:52. | Costa Rica | <5 years | 1992 a 2007 | 3,4 |
| **[88]** | Constenla D, Sinha A, Valencia JE, Gomez E, de la Hoz F, Valenzuela MT, de Quadros CA. Identifying unit costs for use in regional economic evaluation: An illustrative analysis of childhood pneumococcal conjugate vaccine in Latin America and the Caribbean. Rev Panam Salud Pública. 2009; 26:458-468. | Latin-American | <5 years | 2006 | 4 |
| **[89]** | Grupo de Trabajo de SIREVA II Informe Regional de SIREVA II: Organización Panamericana de la Salud. Informe Regional de SIREVA II, **2008**: datos por país y por grupo de edad sobre las características de los aislamientos de *Streptococcus pneumoniae*, *Haemophilus influenzae* y *Neisseria meningitidis* en procesos invasivos, 2008. (Serie Documentos técnicos. Tecnologías esenciales de salud. THS/EV-2009/002). Washington: OPS; 2009. | LAC^b^ | <5 years | 2008 | 1 |
| **[34]** | Castañeda E, Agudelo CI, Regueira M, Corso A, Brandileone MC, Brandão AP, Maldonado A, Hormazabal JC, Martínez IT, Llanes R, Sánchez J, Feris JM, Echániz-Aviles G, Carnalla-Barajas MN, Terrazas MG, Monroy IH, Chamorro G, Weiler N, Camou T, Gabarrot GG, Spadola E, Payares D, Gabastou JM, Di Fabio JL, de la Hoz F; SIREVA II Group. Laboratory-based surveillance of *Streptococcus pneumoniae* invasive disease in children in 10 Latin American countries: a SIREVA II project, 2000-2005. Pediatr Infect Dis J. 2009; 28:e265-270. | Argentina, Brazil, Chile, Colombia, Cuba, DR, Mexico, Paraguay, Uruguay and Venezuela | <6 years | 2000-2005 | 1 |
| **[90]** | Lagos R, Muñoz A, Espinoza A, Dowes A, Ruttimann R, Colindres R. Costos médicos directos de enfermedades neumocócicas invasoras y neumonías con diagnóstico radiológico en niños chilenos. Rev Panam Salud Pública. 2009; 26:101-111. | Chile | <3 years | 2008 | 4 |
| **[62]** | Valenzuela MT, O'Loughlin R, de la Hoz F, Gomez E, Constenla D, Sinha A, Valencia JE, Flannery B, De Quadros CA. The burden of pneumococcal disease among Latin American and Caribbean children: Review of the evidence. Rev Panam Salud Pública. 2009; 25:270-279. | Latin-America | <6 years | 1990-2006 | 4 |
| **[59]** | Azevedo J, Galvão VS, Sant'Anna V, da Matta TF, Cordeiro SM, dos Reis MG, Ko AI, Reis JN, Campos LC: Pneumoccocal carriage profile in Brazilian pediatric patients from the pre-vaccine era. In Presented at: 7th International Symposium on Pneumococci and Pneumococcal Diseases (ISPPD-7). Tel-Aviv; 2010. | Brazil | <5 years | 2009 | 3 |
| **[91]** | Berezin E, Hong T, Markowitz J, Seljan MP. Epidemiology and outcomes of hospitalizations due to pneumonia in young children living in Brazil between 2003 and 2007. Presented at: 6th Annual Meeting of the World Society for Pediatric Infectious Diseases – WSPID. Buenos Aires, Argentina, 18–22 November 2009. | Brazil | <5 years | 2003-2007 | 4 |
| **[92]** | Cedrès A, Sobrero H, Giachetto A, Algorta G, Montano A, Pirez MC. 7-valent pneumococcal conjugate vaccine mass vaccination. Bacterial pneumonia admissions in children before and during the year of intervention. Uruguay. Presented at: 6th Annual Meeting of the World Society for Pediatric Infectious Diseases – WSPID. Buenos Aires, Argentina, 18–22 November 2009. | Uruguay | <5 years | 2005-2009 | 4 |
| **[39]** | Gentile A, Prieto N, Fossati S, Rodriguez M, Sorhouet C, Gagetti P, Moscoloni MA, Lamy P, Regueira M, Corso A, Spn Working Group. Nasopharyngeal carriage (NPC) of *S. pneumoniae* (Spn) among non vaccinated children during winter-summer seasons: first national study in Argentina. Presented at: 6th Annual Meeting of the World Society for Pediatric Infectious Diseases – WSPID. Buenos Aires, Argentina, 18–22 November 2009. | Argentina | <3 years | 2007-2008 | 3 |
| **[93]** | Giglio N., Cane A., Ferreiros E. Evaluation of cost -effectiveness of three vaccination scenarios with pneumococcal conjugated vaccines. Presented at: 6th Annual Meeting of the World Society for Pediatric Infectious Diseases – WSPID. Buenos Aires, Argentina, 18–22 November 2009. | Argentina | <5 years | 2008 | 4 |
| **[94]** | González Ayala S, Vescina C, Agosti M, Morales J, Marone J, Regueira M, Moriconi L. Pneumococcal meningitis at an Argentinean municipality. Presented at: 6th Annual Meeting of the World Society for Pediatric Infectious Diseases – WSPID. Buenos Aires, Argentina, 18–22 November 2009. | Argentina | <5 years | 2008 | 4 |
| **[95]** | Gutierrez S, Boix AI, Gotelo G, Silva L. Bacterial pneumonia. Hospital admission to the Sindicato Medico Uruguay (CASMU) one year before and after introduction of 7-valent pneumococcal vaccine. Presented at: 6th Annual Meeting of the World Society for Pediatric Infectious Diseases – WSPID. Buenos Aires, Argentina, 18–22 November 2009. | Uruguay | <5 years | 2008 | 4 |
| **[36]** | López P, Sierra A, Zapata MA, Vanegas B, De Antonio R, Castrejon MM, Hausdorff WP, Colindres R. Non-typeable *Haemophilus influenzae* and *Streptococcus pneumoniae*: primary causes of acute otitis media in Colombian children. Presented in 13th International Congress on Infectious Diseases (ICID) 2009, USA. | Colombia | <5 years | 2008-2009 | 3 |
| **[52]** | Mendez D, Bolaños R. Nasopharyngeal carriage of *Streptococcus pneumoniae* in healthy children attending daycare centers in Panama. Presented at: 6th Annual Meeting of the World Society for Pediatric Infectious Diseases – WSPID. Buenos Aires, Argentina, 18–22 November 2009. | Panama | <5 years | 2008 | 3 |
| **[2]** | O'Brien KL, Wolfson LJ, Watt JP, Henkle E, Deloria-Knoll M, McCall N, Lee E, Mulholland K, Levine OS, Cherian T. Hib and Pneumococcal Global Burden of Disease Study Team. Burden of disease caused by *Streptococcus pneumoniae* in children younger than 5 years: global estimates. Lancet. 2009; 374:893-902. | World | <5 years | 2000 | 4 |
| **[56]** | Bello González T, Rivera-Olivero IA, Pocaterra L, Spadola E, Araque M, Hermans PWM, De Waard JH. Estado de portador nasofaríngeo de *Streptococcus pneumoniae* en madres e hijos de la población indígena Panare del estado Bolívar, Venezuela. Rev Argent Microbiol.2010; 42: 30-34. | Venezuela | <5 years | 2008 | 3 |
| **[43]** | Franco CM, Andrade AL, Andrade JG, Almeida e Silva S, Oliveira CR, Pimenta FC, Lamaro-Cardoso J, Brandão AP, Almeida SC, Calix JJ, Nahm MH, de Cunto Brandileone MC. Survey of nonsusceptible nasopharyngeal *Streptococcus pneumoniae* isolates in children attending day-care centers in Brazil. Pediatr Infect Dis J. 2010; 29:77-79. | Brazil | <24 months | 2005 | 3 |
| **[96]** | Giglio ND, Cane AD, Micone P, Gentile A. Cost-effectiveness of the CRM-based 7-valent pneumococcal conjugated vaccine (PCV7) in Argentina. Vaccine. 2010; 28:2302-2310. | Argentina | <5 years | 2006 | 4 |
| **[47]** | Parra E, Moreno J, Sanabria O. Detection and serotyping of *Streptococcus pneumoniae* from Nasopharyngeal samples of children using multiplex-PCR before heptavalent pneumococcal vaccination in Popayan Colombia. Presented at: 7th International Symposium on Pneumococci and Pneumococcal Diseases (ISPPD-7), 2010, Tell-Aviv, Israel. | Colombia | <2 years | 2009 | 3 |
| **[97]** | Grupo de Trabajo de SIREVA II Informe Regional de SIREVA II: Organización Panamericana de la Salud. Informe Regional de SIREVA II, 2009: datos por país y por grupo de edad sobre las características de los aislamientos de *Streptococcus pneumoniae, Haemophilus influenzae* y *Neisseria meningitidis* en procesos invasivos. (Serie Documentos técnicos. Tecnologías esenciales de salud. THS/EV-2009/002). Washington: OPS; 2010. | LAC^b^ | <5 years | 2009 | 1 |

^a^ Category: 1 = SIREVA invasive isolates, 2= Non-SIREVA invasive isolates, 3= Non-SIREVA non-invasive isolates, 4=burden disease

^b^ Latin America and the Caribbean (LAC): Argentina, Bolivia, Brazil, Caribbean Epidemiology Center, Chile, Colombia, Costa Rica, Cuba, DR, Ecuador, El Salvador, Guatemala, Honduras, Mexico, Nicaragua, Panama, Paraguay, Peru, Uruguay and Venezuela (<http://new.paho.org/hq/index.php?option=com_content&task=view&id=1077&Itemid=1273&limit=1&limitstart=2&lang=es>)

**37 Studies included only as reference (SIREVA and non SIREVA)**

| **Reference** | **Study** |
| --- | --- |
| **[98]** | Brandileone MC, Vieira VS, Casagrande ST, Zanella RC, Guerra ML, Bokermann S, De Moraes JC, Baldacci ER, Chamone CB, Oliveira MA, De Matos DG, Arruda TM, Coelho MF, D'Avila SM, Dos Santos AR, Di Fabio JL. Prevalence of serotypes and antimicrobial resistance of *Streptococcus pneumoniae* strains isolated from Brazilian children with invasive infections. Pneumococcal Study Group in Brazil for the SIREVA Project. Regional System for Vaccines in Latin America. Microb Drug Resist. 1997;3:141-146. SIREVA |
| **[99]** | Castañeda E, Leal AL, Castillo O, De La Hoz F, Vela MC, Arango M, Trujillo H, Levy A, Gama ME, Calle M, Valencia ML, Parra W, Agudelo N, Mejía GI, Jaramillo S, Montoya F, Porras H, Sánchez A, Saa D, Di Fabio JL, Homma A. Distribution of capsular types and antimicrobial susceptibility of invasive isolates of *Streptococcus pneumoniae* in Colombian children. Microb Drug Resist. 1997; 3:147-152. SIREVA |
| **[100]** | Echániz-Aviles G, Velázquez-Meza ME, Carnalla-Barajas MN, Soto-Noguerón A, Solórzano-Santos F, Pérez Miravete A, Gatica-Marquina R, di Fabio JL. Antimicrobial susceptibilities and capsular types of invasive *Streptococcus pneumoniae* isolated in children in Mexico City. Microb Drug Resist 1997; 3:153-157. SIREVA |
| **[101]** | Hortal M, Algorta G, Bianchi I, Borthagaray G, Cestau I, Camou T, Castro M, De Los Santos M, Diez R, Dell'Acqua L, Galiana A, Giordano A, Giordano P, Lopez-Ghemi G, Milanese N, Mogdasy C, Palacio R, Pedreira W, Pisano A, Pivel L. Capsular type distribution and susceptibility to antibiotics of *Streptococcus pneumoniae* clinical strains isolated from Uruguayan children with systemic infections. Microb Drug Resist. 1997;3:159-164. SIREVA |
| **[102]** | Rossi A, Ruvinsky R, Regueira M, Corso A, Pace J, Gentile A, Di Fabio JL. Distribution of capsular types and penicillin-resistance of strains of *Streptococcus pneumoniae* causing systemic infections in Argentinean children under 5 years of age. *Streptococcus pneumoniae* Working Group. Microb Drug Resist. 1997;3:135-140. SIREVA |
| **[103]** | Kertesz DA, Di Fabio JL, de Cunto Brandileone MC, Castañeda E, Echániz-Aviles G, Heitmann I, Homma A, Hortal M, Lovgren M, Ruvinsky RO, Talbot JA, Weekes J, Spika JS. Invasive *Streptococcus pneumoniae* infection in Latin American children: results of the Pan American Health Organization Surveillance Study. Clin Infect Dis. 1998;26:1355-1361. SIREVA. |
| **[104]** | Tomasz A, Corso A, Severina EP, Echániz-Aviles G, Brandileone MC, Camou T, Castañeda E, Figueroa O, Rossi A, Di Fabio JL. Molecular epidemiologic characterization of penicillin-resistant *Streptococcus pneumoniae* invasive pediatric isolates recovered in six Latin-American countries: an overview. Microb Drug Resist. 1998;4:195-207. SIREVA |
| **[105]** | Levine MM, Lagos R, Levine OS, Heitmann I, Enriquez N, Pinto ME, Alvarez AM, Wu E, Mayorga C, Reyes A. Epidemiology of invasive pneumococcal infections in infants and young children in Metropolitan Santiago, Chile, a newly industrializing country. Pediatr Infect Dis J. 1998; 17:287-293. SIREVA |
| **[106]** | Leal AL, Castañeda E. Susceptibility to antimicrobial agents in isolates of invasive *Streptococcus pneumoniae* in Colombia. Pan Am J Public Health. 1999; 5:157-163. SIREVA |
| **[107]** | Gomez Barreto D, Calderon-Jaimes E, Rodriguez RS, De Los Monteros LEE. Clinical outcome of invasive infections in children caused by highly penicillin-resistant *Streptococcus pneumoniae* compared with infections caused by penicillin-susceptible strains. Arch Med Res. 2000;31:592-596. SIREVA |
| **[108]** | Hortal M, Ruvinsky R, Rossi A, Agudelo CI, Castañeda E, Brandileone C, Camou T, Palacio R, Echániz G, Di Fabio JL. Impacto de *Streptococcus pneumoniae* en las neumonías del niño latinoamericano. Grupo SIREVA-Vigía1. Rev Panam Salud Pública. 2000;8:185-195. SIREVA |
| **[109]** | Hortal M, Lovgren M, de la Hoz F, Agudelo CI, Brandileone MC, Camou T, Casagrande S, Castañeda E, Corso A, Echaniz G, Hormazabal JC, Pace J, Palacio R, Perez-Giffoni G, Ruvinsky R, Di Fabio JL and the PAHO SIREVA-Vigía Study Groups. Antibiotic resistance in Streptococcus pneumoniae in six Latin American countries: biological and therapeutic implications. Microb Drug Resist. 2001;7:391-401. SIREVA |
| **[110]** | Nascimento-Carvalho CM, Lopes AA, Gomes MD, Magalhães MP, Oliveira JR, Vilas-Boas AL, Ferracuti R, Brandileone MC, Guerra ML, Alves NN, Athayde LA, Caldas RM, Barberino MG, Duarte J, Brandão MA, Rocha H, Benguigui Y, Di Fabio JL. Community acquired pneumonia among pediatric outpatients in Salvador, Northeast Brazil, with emphasis on the role of pneumococcus. Braz J Infect Dis. 2001;5:13-20. SIREVA |
| **[111]** | Rey LC, Wolf B, Moreira JL, Milatovic D, Verhoef J, Farhat CK. Antimicrobial susceptibility and serotypes of nasopharyngeal *Streptococcus pneumoniae* in children with pneumonia and in children attending day-care centres in Fortaleza, Brazil. Int J Antimicrob Agents. 2002;20:86-92. |
| **[112]** | Ruvinsky R, Gentile A, Regueira M, Corso A. Infecciones invasivas por *Streptococcus pneumoniae:* estudio epidemiológico e importancia del desarrollo de un sistema de vigilancia. Arch Argent Pediatr. 2002;100:31-43. SIREVA |
| **[113]** | Camou T, Palacio R, Di Fabio JL, Hortal M. Invasive pneumococcal diseases in Uruguayan children: Comparison between serotype distribution and conjugate vaccine formulations. Vaccine. 2003;21:2102-2105. SIREVA |
| **[114]** | Grupo Multifuncional de Neumonías Rev Peru Med Exp Salud Pública. 2003;20:150-155. SIREVA |
| **[115]** | Magalhaes AP, Pinto AS. Antimicrobial resistance and serotyping *Streptococcus pneumoniae* isolated from pediatric patients in Bello Horizonte, MG, Brazil. Braz J Microbiol. 2003;34:210-212. SIREVA |
| **[116]** | Moreno J, Phandanouvong V, Castañeda E. Vigilancia molecular de aislamientos invasores de *Streptococcus pneumoniae* resistentes a la penicilina en niños colombianos menores de 5 años. Biomédica. 2004;24:296-301. SIREVA |
| **[117]** | Ochoa TJ, Rupa R, Guerra H, Hernandez H, Chaparro E, Tamariz J, Wanger A, Mason EO Jr. Penicillin resistance and serotypes/ serogroups of *S. pneumoniae* in nasopharyngeal carrier children younger than 2 years in Lima, Peru. Diagn Microbiol Infect Dis. 2005; 52:59-64. |
| **[118]** | Zemlicková H, Crisóstomo MI, Brandileone MC, Camou T, Castañeda E, Corso A, Echániz-Aviles G, Pásztor M, Tomasz A. Serotypes and clonal types of penicillin-susceptible *Streptococcus pneumoniae* causing invasive disease in children in five Latin American countries. Microb Drug Resist. 2005;11:195-204. SIREVA |
| **[119]** | Agudelo CI, Moreno J, Sanabria OM, Ovalle MV, Di Fabio JL, Castañeda E. *Streptococcus pneumoniae*: Evolución de los serotipos y los patrones de susceptibilidad antimicrobiana en aislamientos invasores en 11 años de vigilancia en Colombia (1994 –2004). Grupo colombiano de trabajo en *S. pneumoniae* Biomédica.2006;26:234-249. SIREVA |
| **[120]** | Bricks LF, Berezin E. Impact of pneumococcal conjugate vaccine on the prevention of invasive pneumococcal diseases. J Pediatr (Rio J). 2006; 82(3 Suppl):S67-S74. SIREVA |
| **[121]** | Lagos RM, Muñoz AE, Levine MM. Prevalence of pneumococcal bacteremia among children <36 months of age presenting with moderate fever to pediatric emergency rooms of the Metropolitan Region (Santiago), Chile. Human Vaccines. 2006;2:129-133. SIREVA. |
| **[122]** | Tique V, Alvis N, Parodi R, Bustos A, Mattar S. Meningitis agudas en Córdoba, Colombia 2002 – 2004. Rev Salud Pública (Bogota). 2006;8 Suppl 1:33-46. SIREVA |
| **[123]** | Tregnaghi M, Ceballos A, Rüttimann R, Ussher J, Tregnaghi P, Peeters P, Hausdorff WP. Active epidemiologic surveillance of pneumonia and invasive pneumococcal disease in ambulatory and hospitalized infants in Cordoba, Argentina. Pediatr Infect Dis J. 2006;25:370-372. |
| **[124]** | Ferrari Castilla AM, Pirez G MC, Martínez A A, Algorta R G, Chamorro V F, Guala B MJ, Zabala Ch C, Giachetto L G, Montano L A. Etiología de la neumonía bacteriana adquirida en la comunidad en niños hospitalizados. Uruguay 1998-2004. Rev Chilena Infectol. 2007;24:40-47. SIREVA |
| **[125]** | Lovgren M, Talbot JA, Brandileone MC, Casagrande ST, Agudelo CI, Castañeda E, Regueira M, Corso A, Heitmann I, Maldonado A, Echániz-Aviles G, Soto-Noguerón A, Hortal M, Camou T, Gabastou JM, Di Fabio JL; SIREVA Study Group. Evolution of an international external quality assurance model to support investigation of *Streptococcus pneumoniae*, developed for the SIREVA Project in Latin America, from 1993 to 2005. J Clin Microbiol. 2007;45:3184-3190. SIREVA |
| **[126]** | Vieira A, Gomes, Rolo M, Eudes J, Bello EJM, Figueiredo R. *Streptococcus pneumoniae*: estudo das cepas isoladas de liquor. J Pediatr. (Rio J.) 2007; 83:71-78. SIREVA |
| **[57]** | Berezin EN, Cardenuto MD, Ferreira LL, Otsuka M, Guerra ML, Brandileone MC. Distribution of *Streptococcus pneumoniae* serotypes in nasopharyngeal carriage and in invasive pneumococcal disease in Sao Paulo, Brazil. Pediatr Infect Dis J. 2007;267:643-645. SIREVA (invasive isolates) |
| **[127]** | Abarca VK, Vergara FR, Tassara PE, Ibanez W, I, Garcia BC, Potin SM. Invasive pneumococcal disease and consolidated pneumonia in infants: one year of surveillance in three Chilean health care centers. Rev Chilena Infectol. 2008; 25:97-103. SIREVA |
| **[128]** | Gabastou JM, Agudelo CI, Brandileone MC, Castañeda E, de Lemos AP, Di Fabio JL. Caracterización de aislamientos invasivos de *S. pneumoniae, H. influenzae y N. meningitidis* en América Latina y el Caribe: SIREVA II, 2000-2005. Rev Panam Salud Pública. 2008;24:1-15. SIREVA |
| **[129]** | Hortal M, Sehabiague G, Camou T, Iraola I, Estevan M, Pujadas M. Pneumococcal pneumonia in hospitalized Uruguayan children and potential prevention with different vaccine formulations. J Pediatr. 2008;152:850-853. SIREVA |
| **[130]** | Mayoral C, Baroni MR, Giani R, Virgolini S, Zurbriggen L, Regueira M. Distribución de serotipos de *Streptococcus pneumoniae* aislados de infecciones invasoras en el Hospital de Niños de Santa Fe. Rev Argent Microbiol. 2008; 40:13-16. SIREVA |
| **[131]** | Pírez García MC, Giachetto Larraz G, Romero Rostagno C, Zabala Chain C, Algorta Rusiñol G, Montano Lotito A, Ferrari Castilla AM. Neumonía neumocócica invasiva en niños de 0 a 24 meses: ¿influye la resistencia bacteriana en la evolución? An Pediatr (Barc). 2008;69:205-209. SIREVA |
| **[132]** | Agudelo CI, Castañeda E, Corso A, Regueira M, Brandileone MC, Brandão AP, Maldonado A, Hormazabal JC, Tamargo I, Echániz-Aviles G, Soto A, Viveros MG, Hernández I, Chamorro G, Weiler N, Sánchez J, Feris JM, Camou T, García G, Spadola E, Payares D, Gabastou JM, Di Fabio JL; Grupo Sireva II. Resistencia a antibióticos no betalactámicos de aislamientos invasores de *Streptococcus pneumoniae* en niños latinoamericanos. SIREVA II, 2000–2005. Rev Panam Salud Pública. 2009;25:305–313. SIREVA |
| **[133]** | Mantese OC. Prevalence of serotypes and antimicrobial resistance of invasive strains of pneumococcus in children: Analysis of 9 years. J Pediatr (Rio J). 2009; 85:495-502. SIREVA |
| **[134]** | Lagos R, Muñoz A, Valenzuela MT, Heitmann I, Levine MM: Population-based surveillance for hospitalized and ambulatory pediatric invasive pneumococcal disease in Santiago, Chile. Pediatr Infect Dis J 2002, 21:1115–1123. |
| **[135]** | Dickinson FO, Pérez AE: Bacterial meningitis in children and adolescents: an observational study based on the national surveillance system. BMC Infect Dis 2005, 5:103. |
