# Supplementary material for: Streptococcus pneumoniae serotype 19A in Latin America and the Caribbean: a systematic review and meta-analysis, 1990–2010
Source: BMC Infect Dis. 2012 May 28;12:124. doi: 10.1186/1471-2334-12-124 (PMC3475047; doi:10.1186/1471-2334-12-124)
Supplement: Additional file 3 — a- Streptococcus pneumoniae. Number of invasive isolates by serotype and country presented by vaccine type (VT) (PCV7, PCV10, PCV13) and non vaccine type (NVT). SIREVA, 1993–2009 and non SIREVA, 1989–2008. b - Streptococcus pneumoniae. Serotypes invasive isolates, < 6 years old, SIREVA, 20 countries*, 2000–2005. c - Streptococcus pneumoniae. Serotypes invasive isolates, < 5 years old, SIREVA, 20 countries*, 2006–2009 [27-33,75,82,85,86,89,97]. [file 1471-2334-12-124-S3.docx]

**Supplement 3**

**Supplement 3a. *Streptococcus pneumoniae*. Number of invasive isolates by serotype and country presented by vaccine type (VT) (PCV7, PCV10, PCV13) and non vaccine type (NVT). SIREVA, 1993-2009 and non SIREVA, 1989-2008**

**SIREVA, 1993-2009. *Streptococcus pneumoniae*. Total invasive isolates**

| **Country**  (year of isolate) [reference] | **VT** | | | | | | | | | | | | | **NVT** | **Total** |
| --- | --- | --- | --- | --- | --- | --- | --- | --- | --- | --- | --- | --- | --- | --- | --- |
|  | **PCV13** | | | | | | | | | | | | |  |  |
|  | **PCV10** | | | | | | | | | |  | | |  |  |
|  | **PCV7** | | | | | | |  | | |  |  |  |  |  |
|  | **4** | **6B** | **9V** | **14** | **18C** | **19F** | **23F** | **1** | **5** | **7F** | **3** | **6A** | **19A** |  |  |
|  | **n** | | | | | | | | | | | | | | |
| Argentina  (1993-2009) | 20 | 97 | 88 | 812 | 81 | 66 | 80 | 268 | 368 | 120 | 44 | 137 | **100** | 411 | **2,692** |
| Bolivia  (2000-2009) | 1 | 22 | 1 | 44 | 6 | 12 | 2 | 10 | 8 | 5 | 0 | 3 | **5** | 41 | **160** |
| Brazil  (1993-2009) | 72 | 346 | 135 | 1443 | 257 | 207 | 206 | 246 | 190 | 70 | 93 | 312 | **153** | 532 | **4,262** |
| CAREC^a^  (2000-2009) | 0 | 30 | 1 | 53 | 9 | 8 | 15 | 0 | 0 | 2 | 1 | 9 | **2** | 18 | **148** |
| Chile  (1993-2009) | 73 | 226 | 47 | 987 | 226 | 203 | 118 | 284 | 196 | 146 | 49 | 213 | **161** | 688 | **3,617** |
| Colombia  (1993-2009) | 25 | 118 | 44 | 551 | 136 | 94 | 139 | 150 | 100 | 27 | 32 | 169 | **28** | 206 | **1,819** |
| Costa Rica  (2000-2009) | 3 | 5 | 3 | 22 | 0 | 4 | 3 | 0 | 0 | 1 | 4 | 3 | **3** | 12 | **63** |
| Cuba  (2000-2009) | 10 | 119 | 36 | 110 | 140 | 125 | 46 | 80 | 22 | 63 | 21 | 32 | **31** | 64 | **899** |
| DR^b^  (2000-2009) | 10 | 60 | 11 | 228 | 16 | 25 | 38 | 33 | 15 | 3 | 17 | 31 | **14** | 43 | **544** |
| Ecuador  (2000-2009) | 2 | 21 | 10 | 61 | 2 | 8 | 8 | 21 | 14 | 2 | 5 | 9 | **5** | 47 | **215** |
| El Salvador  (2000-2009) | 0 | 2 | 8 | 9 | 5 | 0 | 2 | 2 | 0 | 2 | 4 | 7 | **5** | 6 | **52** |
| Guatemala  (2000-2009) | 3 | 4 | 9 | 13 | 3 | 4 | 16 | 17 | 5 | 5 | 1 | 6 | **11** | 21 | **118** |
| Honduras  (2000-2009) | 0 | 2 | 0 | 1 | 0 | 0 | 1 | 0 | 0 | 0 | 0 | 0 | **0** | 7 | **11** |
| Mexico  (1993-2009) | 21 | 122 | 52 | 153 | 42 | 200 | 201 | 25 | 19 | 20 | 33 | 128 | **91** | 356 | **1,463** |
| Nicaragua  (2000-2009) | 1 | 3 | 0 | 8 | 1 | 0 | 4 | 1 | 4 | 0 | 1 | 1 | **2** | 17 | **43** |
| Panama  (2000-2009) | 8 | 38 | 4 | 27 | 9 | 17 | 8 | 1 | 13 | 1 | 8 | 12 | **15** | 33 | **194** |
| Paraguay  (2000-2009) | 14 | 52 | 26 | 288 | 5 | 11 | 19 | 74 | 112 | 23 | 5 | 16 | **17** | 122 | **784** |
| Peru  (2000-2009) | 3 | 30 | 3 | 93 | 3 | 15 | 7 | 6 | 15 | 0 | 2 | 17 | **8** | 47 | **249** |
| Uruguay  (1993-2009) | 11 | 39 | 34 | 390 | 24 | 25 | 17 | 169 | 181 | 69 | 62 | 41 | **44** | 104 | **1,210** |
| Venezuela  (2000-2009)^b^ | 9 | 80 | 9 | 178 | 21 | 23 | 18 | 35 | 51 | 19 | 21 | 19 | **38** | 20 | **541** |
| **Sub-Total**  **[75,82,85,86,**  **89,97]** | **286** | **1,416** | **521** | **5,471** | **986** | **1,047** | **948** | **1,422** | **1,313** | **578** | **403** | **1,165** | **733** | **2,795** | **19,084** |
| **%** | **1.5** | **7.4** | **2.7** | **28.7** | **5.2** | **5.5** | **5.0** | **7.5** | **6.9** | **3.0** | **2.1** | **6.1** | **3.8** | **14.6** | **100.0** |
| **Rank** | **13** | **3** | **11** | **1** | **8** | **6** | **7** | **2** | **4** | **10** | **12** | **5** | **9** |  |  |

^a^ CAREC: Caribbean Epidemiology Centre

^b^DR: Dominican Republic

**Non-SIREVA, 1989-2008*, Streptococcus pneumoniae*. Total invasive isolates.**

| **Country**  (year of isolate) [reference] | **VT** | | | | | | | | | | | | | **NVT** | **Total** |
| --- | --- | --- | --- | --- | --- | --- | --- | --- | --- | --- | --- | --- | --- | --- | --- |
|  | **PCV13** | | | | | | | | | | | | |  |  |
|  | **PCV10** | | | | | | | | | |  | | |  |  |
|  | **PCV7** | | | | | | |  | | |  |  |  |  |  |
|  | **4** | **6B** | **9V** | **14** | **18C** | **19F** | **23F** | **1** | **5** | **7F** | **3** | **6A** | **19A** |  |  |
|  | **n** | | | | | | | | | | | | | | |
| Argentina  (1999-2002) [27] | 1 | 17 | 4 | 70 | 11 | 4 | 1 | 13 | 13 | 4 | 1 | 3 | **0** | 11 | **153** |
| Brazil  (1989-1993) [28] |  | 8 |  | 12 |  | 2 | 1 | 3 | 8 |  |  |  | **2** | 13 | **49** |
| Brazil  (1995-1999) [29] | 6 | 17 | 0 | 40 | 9 | 10 | 7 | 0 | 4 | 4 | 3 | 12 | **2** | 31 | **145** |
| Chile  (1994-2004) [30] | 2 | 16 | 5 | 10 | 4 | 12 | 12 | 20 | 12 | 2 |  | 6 | **3** | 24 | **128** |
| Chile  (1995-1997) [31] | 1 | 6 |  | 3 | 1 | 1 | 1 | 3 | 4 | 2 |  | 1 | **1** | 6 | **30** |
| Mexico (1992-1993) [32] | 1 | 13 | 7 | 11 | 3 | 6 | 24 | 1 | 1 | 1 | 1 | 8 | **11** | 32 | **120** |
| Colombia  (2006)  [33] | 1 | 3 |  | 11 |  | 3 | 1 | 2 |  |  |  |  | **1** | 1 | **20** |
| **Sub Total** | **12** | **80** | **16** | **157** | **28** | **38** | **47** | **42** | **43** | **13** | **5** | **30** | **20** | **118** | **649** |
| **%** | **1.8** | **12.3** | **2.5** | **24.2** | **4.3** | **5.9** | **7.2** | **6.5** | **6.6** | **2.0** | **0.8** | **4.6** | **3.1** | **18.2** | **100.0** |
| **Rank** | **12** | **2** | **10** | **1** | **8** | **6** | **3** | **5** | **4** | **11** | **13** | **7** | **9** |  |  |
|  | | | | | | | | | | | | | | | |
| **n Total** | **298** | **1,496** | **537** | **5,628** | **1,014** | **1,085** | **995** | **1,464** | **1,356** | **591** | **408** | **1,195** | **753** | **2,913** | **19,733** |
| **%** | **1.5** | **7.6** | **2.7** | **28.5** | **5.1** | **5.5** | **5.0** | **7.4** | **6.9** | **3.0** | **2.1** | **6.1** | **3.8** | **14.8** | **100.0** |
| **Rank** | **13** | **2** | **11** | **1** | **7** | **6** | **8** | **3** | **4** | **10** | **12** | **5** | **9** |  |  |

## **Supplement 3b. *Streptococcus pneumoniae*. Serotypes invasive isolates, < 6 years old, SIREVA, 20 countries*, 2000-2005**

***Streptococcus pneumoniae*. Total invasive isolates, < 6 years SIREVA, 20 countries, 2000-2005 presented by vaccine type (VT) (PCV7, PCV10, PCV13) and non vaccine type (NVT)**

| **Country** | **VT** | | | | | | | | | | | | | **NTV** | **Total** |
| --- | --- | --- | --- | --- | --- | --- | --- | --- | --- | --- | --- | --- | --- | --- | --- |
|  | **PCV13** | | | | | | | | | | | | |  |  |
|  | **PCV10** | | | | | | | | | |  | | |  |  |
|  | **PCV7** | | | | | | |  | | |  |  |  |  |  |
|  | **4** | **6B** | **9V** | **14** | **18C** | **19F** | **23F** | **1** | **5** | **7F** | **3** | **6A** | **19A** |  |  |
|  | **n** | | | | | | | | | | | | | | |
| Argentina | 8 | 66 | 32 | 298 | 34 | 22 | 32 | 94 | 128 | 37 | 18 | 21 | **33** | 113 | 936 |
| Bolivia | 1 | 13 | 1 | 26 | 3 | 11 | 2 | 6 | 7 | 3 | 0 | 2 | **3** | 30 | 108 |
| Brazil | 35 | 215 | 55 | 702 | 120 | 89 | 88 | 110 | 84 | 41 | 36 | 76 | **71** | 241 | 1,963 |
| CAREC^a^ | 0 | 20 | 0 | 47 | 3 | 6 | 13 | 0 | 0 | 0 | 1 | 4 | **1** | 15 | 110 |
| Chile | 42 | 114 | 28 | 470 | 140 | 106 | 53 | 127 | 104 | 83 | 15 | 74 | **81** | 457 | 1,894 |
| Colombia | 5 | 64 | 21 | 231 | 28 | 17 | 47 | 64 | 38 | 6 | 13 | 42 | **6** | 65 | 647 |
| Costa Rica | 0 | 1 | 0 | 0 | 0 | 0 | 0 | 0 | 0 | 0 | 0 | 0 | **0** | 2 | 3 |
| Cuba | 9 | 109 | 35 | 100 | 136 | 119 | 41 | 78 | 21 | 60 | 19 | 27 | **28** | 60 | 842 |
| DR^b^ | 8 | 43 | 6 | 171 | 13 | 16 | 24 | 23 | 13 | 3 | 12 | 19 | **8** | 20 | 379 |
| Ecuador | 0 | 7 | 2 | 17 | 1 | 3 | 3 | 4 | 3 | 0 | 0 | 3 | **0** | 12 | 55 |
| El Salvador | 0 | 2 | 2 | 3 | 3 | 0 | 1 | 1 | 0 | 0 | 2 | 2 | **3** | 4 | 23 |
| Guatemala | 2 | 4 | 5 | 9 | 3 | 4 | 13 | 17 | 5 | 3 | 1 | 4 | **4** | 17 | 91 |
| Honduras | 0 | 0 | 0 | 0 | 0 | 0 | 0 | 0 | 0 | 0 | 0 | 0 | **0** | 3 | 3 |
| Mexico | 7 | 83 | 30 | 69 | 24 | 124 | 95 | 15 | 4 | 12 | 16 | 31 | **42** | 176 | 728 |
| Nicaragua | 1 | 3 | 0 | 8 | 1 | 0 | 3 | 1 | 3 | 0 | 1 | 1 | **1** | 16 | 39 |
| Panama | 6 | 19 | 2 | 10 | 2 | 11 | 4 | 0 | 3 | 0 | 7 | 7 | **7** | 23 | 101 |
| Paraguay | 9 | 36 | 19 | 157 | 5 | 6 | 13 | 38 | 74 | 13 | 4 | 11 | **12** | 80 | 477 |
| Peru | 1 | 11 | 3 | 56 | 2 | 2 | 2 | 3 | 12 | 0 | 1 | 11 | **3** | 36 | 143 |
| Uruguay | 7 | 24 | 20 | 183 | 11 | 12 | 4 | 78 | 101 | 25 | 31 | 12 | **18** | 49 | 575 |
| Venezuela | 6 | 56 | 6 | 151 | 17 | 11 | 14 | 29 | 38 | 12 | 18 | 12 | **24** | 13 | 407 |
| **Total** | **147** | **890** | **267** | **2,708** | **546** | **559** | **452** | **688** | **638** | **298** | **195** | **359** | **345** | **1,432** | **9,524** |
| **%** | **1.5** | **9.3** | **2.8** | **28.4** | **5.7** | **5.9** | **4.7** | **7.2** | **6.7** | **3.1** | **2.0** | **3.8** | **3.6** | **15.0** |  |
| **Rank** | **13** | **2** | **11** | **1** | **6** | **5** | **7** | **3** | **4** | **10** | **12** | **8** | **9** |  |  |

^a^ Caribbean Epidemiology Centre

^b^Dominican Republic

**Reference [82]**

## **Supplement 3c. *Streptococcus pneumoniae*. Serotypes invasive isolates, < 5 years old, SIREVA, 20 countries*, 2006-2009.**

***Streptococcus pneumoniae*. Total invasive isolates, < 5 years SIREVA, 20 countries, 2006-2009, presented by vaccine type (VT) (PCV7, PCV10, PCV13) and non vaccine type (NVT)**

| **Country** | VT | | | | | | | | | | | | | **NVT** | **Total** |
| --- | --- | --- | --- | --- | --- | --- | --- | --- | --- | --- | --- | --- | --- | --- | --- |
|  | PCV13 | | | | | | | | | | | | |  |  |
|  | PCV10 | | | | | | | | | |  | | |  |  |
|  | PCV7 | | | | | | |  | | |  |  |  |  |  |
|  | **4** | **6B** | **9V** | **14** | **18C** | **19F** | **23F** | **1** | **5** | **7F** | **3** | **6A** | **19A** |  |  |
|  | **n** | | | | | | | | | | | | |  |  |
| Argentina | 6 | 31 | 23 | 186 | 29 | 19 | 16 | 94 | 98 | 38 | 21 | 27 | **37** | 125 | 750 |
| Bolivia | 0 | 9 | 0 | 18 | 3 | 1 | 0 | 4 | 1 | 2 | 0 | 1 | **2** | 11 | 52 |
| Brazil | 19 | 131 | 27 | 442 | 48 | 64 | 56 | 18 | 26 | 21 | 33 | 52 | **40** | 119 | 1096 |
| CAREC^a^ | 0 | 10 | 1 | 6 | 6 | 2 | 2 | 0 | 0 | 2 | 0 | 5 | **1** | 3 | 38 |
| Chile | 24 | 112 | 14 | 427 | 67 | 80 | 52 | 91 | 34 | 44 | 17 | 82 | **61** | 157 | 1262 |
| Colombia | 6 | 54 | 12 | 151 | 78 | 31 | 24 | 44 | 13 | 7 | 13 | 29 | **18** | 69 | 549 |
| Costa Rica | 3 | 4 | 3 | 22 | 0 | 4 | 3 | 0 | 0 | 1 | 4 | 3 | **3** | 10 | 60 |
| Cuba | 1 | 10 | 1 | 10 | 4 | 6 | 5 | 2 | 1 | 3 | 2 | 5 | **3** | 4 | 57 |
| DR^b^ | 2 | 17 | 5 | 57 | 3 | 9 | 14 | 10 | 2 | 0 | 5 | 12 | **6** | 23 | 165 |
| Ecuador | 2 | 14 | 8 | 44 | 1 | 5 | 5 | 17 | 11 | 2 | 5 | 6 | **5** | 35 | 160 |
| El Salvador | 0 | 0 | 6 | 6 | 2 | 0 | 1 | 1 | 0 | 2 | 2 | 5 | **2** | 2 | 29 |
| Guatemala | 1 | 0 | 4 | 4 | 0 | 0 | 3 | 0 | 0 | 2 | 0 | 2 | **7** | 4 | 27 |
| Honduras | 0 | 2 | 0 | 1 | 0 | 0 | 1 | 0 | 0 | 0 | 0 | 0 | **0** | 4 | 8 |
| Mexico | 2 | 39 | 6 | 37 | 6 | 48 | 33 | 2 | 4 | 4 | 9 | 21 | **21** | 77 | 309 |
| Nicaragua | 0 | 0 | 0 | 0 | 0 | 0 | 1 | 0 | 1 | 0 | 0 | 0 | **1** | 1 | 4 |
| Panama | 2 | 19 | 2 | 17 | 7 | 6 | 4 | 1 | 10 | 1 | 1 | 5 | **8** | 10 | 93 |
| Paraguay | 5 | 16 | 7 | 131 | 0 | 5 | 6 | 36 | 38 | 10 | 1 | 5 | **5** | 42 | 307 |
| Peru | 2 | 19 | 0 | 37 | 1 | 13 | 5 | 3 | 3 | 0 | 1 | 6 | **5** | 11 | 106 |
| Uruguay | 2 | 15 | 6 | 85 | 5 | 3 | 3 | 47 | 28 | 27 | 15 | 7 | **13** | 27 | 283 |
| Venezuela | 3 | 24 | 3 | 27 | 4 | 12 | 4 | 6 | 13 | 7 | 3 | 7 | **14** | 7 | 134 |
| **n Total** | **80** | **526** | **128** | **1,708** | **264** | **308** | **238** | **376** | **283** | **173** | **132** | **280** | **252** | **741** | **5,489** |
| **%** | **1.5** | **9.6** | **2.3** | **31.1** | **4.8** | **5.6** | **4.3** | **6.9** | **5.2** | **3.2** | **2.4** | **5.1** | **4.6** | **13.5** |  |
| **Rank** | **13** | **2** | **12** | **1** | **7** | **4** | **9** | **3** | **5** | **10** | **11** | **6** | **8** |  |  |

^a^ Caribbean Epidemiology Centre

^b^Dominican Republic

**References [85,86,89,97]**
