# Supplementary material for: Streptococcus pneumoniae serotype 19A in Latin America and the Caribbean: a systematic review and meta-analysis, 1990–2010
Source: BMC Infect Dis. 2012 May 28;12:124. doi: 10.1186/1471-2334-12-124 (PMC3475047; doi:10.1186/1471-2334-12-124)
Supplement: Additional file 4 — Streptococcus pneumoniae, serotype prevalence in three time periods of SIREVA results presented by vaccine type (VT) (PCV7, PCV10, PCV13) and non vaccine type (NVT).[75,82,85,86,89,97]. [file 1471-2334-12-124-S4.docx]

## **Supplement 4**

## ***Streptococcus pneumoniae,* serotype prevalence in three time periods of SIREVA results presented by vaccine type (VT) (PCV7, PCV10, PCV13) and non vaccine type (NVT)**

| **Vaccine** | **Serotype** | **1993-1999^a^**  [75] | | | **2000-2005^b^**  [82] | | | **2006-2009^b^**  [85,86,89,97] | | |
| --- | --- | --- | --- | --- | --- | --- | --- | --- | --- | --- |
|  |  | **n= 4,071** | | | **n= 9,524** | | | **n= 5,489** | | |
|  |  | **%** | **95% CI** | | **%** | **95% CI** | | **%** | **95% CI** | |
| **PCV7** | 4 | 1.4 | 0.7 | 2.2 | 1.5 | 0.9 | 2.1 | 1.5 | 1.1 | 1.9 |
|  | 6B | - | - | - | 9.3 | 6.3 | 12.4 | 9.6 | 6.5 | 12.6 |
|  | 9V | 3.1 | 2.1 | 4.1 | 2.8 | 1.9 | 3.8 | 2.3 | 1.0 | 3.6 |
|  | **14^c^** | 25.9 | 18.9 | 32.9 | **28.4** | **22.3** | **34.6** | **31.1** | **26.5** | **35.7** |
|  | **18C^c^** | 4.3 | 2.7 | 6.0 | **5.7** | **4.0** | **7.5** | **4.8** | **2.5** | **7.2** |
|  | 19F | 4.4 | 2.8 | 6.0 | 5.9 | 3.8 | 8.0 | 5.6 | 3.5 | 7.7 |
|  | 23F | 6.3 | 1.7 | 11.0 | 4.7 | 2.9 | 6.5 | 4.3 | 2.8 | 5.8 |
| **PCV10** | 1 | 8.8 | 5.3 | 12.3 | 7.2 | 5.1 | 9.4 | 6.9 | 4.3 | 9.4 |
|  | **5^c^** | 9.6 | 5.8 | 13.5 | **6.7** | **4.4** | **9.0** | **5.2** | **3.0** | **7.4** |
|  | 7F | 2.6 | 1.2 | 4.1 | 3.1 | 2.2 | 4.0 | 3.2 | 2.4 | 3.9 |
| **PCV13** | 3 | 1.9 | 0.6 | 3.1 | 2.0 | 1.0 | 3.1 | 2.4 | 1.5 | 3.3 |
|  | **6A^c^** | 12.9 | 9.4 | 16.5 | **3.8** | **2.7** | **4.8** | **5.1** | **3.6** | **6.6** |
|  | **19A** | **3.3** | **1.8** | **4.9** | **3.6** | **2.3** | **4.9** | **4.6** | **3.4** | **5.8** |
| NVT | Others | 15.3 | 10.9 | 19.7 | 15.0 | 5.0 | 25.1 | 13.5 | 10.6 | 16.4 |

^a^ 6 countries: Argentina, Brazil, Chile, Colombia, Mexico and Uruguay. 6A/6B together

^b^ 20 countries

^c^ p<0.05

- No data

## ***Streptococcus pneumoniae* serotype 19A, prevalence by region (SIREVA) - Average cases by study period (2000-2005 vs. 2006 – 2009)**

| **Region^a^** | **2000-2005 [82]** | | | | **2006-2009 [85,86,89,97]** | | | | **p value** |
| --- | --- | --- | --- | --- | --- | --- | --- | --- | --- |
|  | **Average cases/year** | | **Total** | | **Average cases/year** | | **Total** | |  |
|  | **19A** | **All serotypes** | **19A** | **All serotypes** | **19A** | **All serotypes** | **19A** | **All serotypes** |  |
|  | **n**  **(%)** | **n** | **n**  **(%)** | **n** | **n**  **(%)** | **n** | **n**  **(%)** | **n** |  |
| Andean | 6 (2.9) | 209 | 33 (2.6) | 1,252 | 11 (4.6) | 237 | 42 (4.4) | 949 | 0.02 |
| South Cone | 25 (3.8) | 665 | 147 (3.7) | 3,990 | 30 (4.5) | 664 | 118 (4.4) | 2,654 | 0.12 |
| Brazil | 12 (3.7) | 327 | 71 (3.6) | 1,963 | 10 (3.6) | 274 | 40 (3.6) | 1,096 | 0.96 |
| Mexico | 7 (5.8) | 121 | 42 (5.8) | 728 | 5 (6.5) | 77 | 21 (6.8) | 309 | 0.7 |
| CA and Caribbean | 9 (3.4) | 265 | 52 (3.3) | 1,591 | 8 (6.7) | 120 | 31 (6.4) | 481 | 0.002 |
| **Total** | **58 (3.7)** | **1,587** | **345 (3.6)** | **9,524** | **63 (4.6)** | **1,372** | **252 (4.6)** | **5,489** | **0.003** |

^a^ Region

Andean: Colombia, Ecuador, Peru, Venezuela

South Cone: Argentina, Bolivia, Chile, Paraguay, Uruguay

Central America and Caribbean: Caribbean Epidemiology Center (CAREC), Costa Rica, Cuba, El Salvador, Dominican Republic, Guatemala, Honduras, Nicaragua, Panama
