# Supplementary material for: Streptococcus pneumoniae serotype 19A in Latin America and the Caribbean: a systematic review and meta-analysis, 1990–2010
Source: BMC Infect Dis. 2012 May 28;12:124. doi: 10.1186/1471-2334-12-124 (PMC3475047; doi:10.1186/1471-2334-12-124)
Supplement: Additional file 5 — Streptococcus pneumoniae. Serotype distribution for invasive isolates by age group <2 years and 2–5 years. SIREVA data, 2007–2009 [86,89,97]. [file 1471-2334-12-124-S5.docx]

**Supplement 5**

***Streptococcus pneumoniae.* Serotype distribution for invasive isolates by age group**

**<2 years and 2-5 years. SIREVA data, 2007-2009.**

| **Serotypes** | **Children <2 years** | | **Children 2-5 years** | | **p-value** |
| --- | --- | --- | --- | --- | --- |
|  | **n** | **%** | **n** | **%** |  |
| 1 | 139 | 4.3 | 133 | 13.8 | < 0.001* |
| 3 | 69 | 2.1 | 31 | 3.2 | 0.06 |
| 4 | 42 | 1.3 | 10 | 1.0 | 0.5 |
| 5 | 134 | 4.2 | 59 | 6.1 | 0.01* |
| 6A | 180 | 5.6 | 42 | 4.3 | 0.12 |
| 6B | 328 | 10.2 | 65 | 6.7 | 0.001* |
| 7F | 106 | 3.3 | 35 | 3.6 | 0.62 |
| 9V | 70 | 2.2 | 22 | 2.3 | 0.8 |
| 14 | 1,086 | 33.8 | 271 | 28.1 | < 0.001* |
| 18C | 114 | 3.5 | 38 | 3.9 | 0.57 |
| 19A | 158 | 4.9 | 49 | 5.1 | 0.84 |
| 19F | 165 | 5.1 | 55 | 5.7 | 0.49 |
| 23F | 143 | 4.4 | 42 | 4.3 | 0.89 |
| Others | 481 | 15.0 | 114 | 11.8 | 0.01* |
| **Total** | **3,215** | **100.0** | **966** | **100.0** |  |

*****Statistically significant

**References [86,89,97]**
