# Supplementary material for: Streptococcus pneumoniae serotype 19A in Latin America and the Caribbean: a systematic review and meta-analysis, 1990–2010
Source: BMC Infect Dis. 2012 May 28;12:124. doi: 10.1186/1471-2334-12-124 (PMC3475047; doi:10.1186/1471-2334-12-124)
Supplement: Additional file 6 — a- Streptococcus pneumoniae serotype 19A. Meningitis, non-meningitis in ten countries. SIREVA data, 2000–2009. b - Streptococcus pneumoniae serotype 19A. Non-meningitis and pneumoniae cases in 20 countries. SIREVA data, 2006–2007. c - Streptococcus pneumoniae. Meningitis isolates by country and serotype presented by vaccine type (VT) (PCV7, PCV10, PCV13) and non vaccine type (NVT). SIREVA data, 2000–2009. d - Streptococcus pneumoniae. Non-meningitis isolates by country and serotype presented by vaccine type (VT) (PCV7, PCV10, PCV13) and non vaccine type (NVT). SIREVA data, 2000–2009 [34,82,85,86,89,97]. [file 1471-2334-12-124-S6.docx]

## **Supplement 6**

## **Supplement 6a. *Streptococcus pneumoniae* serotype 19A. Meningitis, non-meningitis in ten countries.**

## **SIREVA data, 2000-2009**

| Country | Meningitis | | |  | Non-meningitis | | p |
| --- | --- | --- | --- | --- | --- | --- | --- |
|  | Total | Serotype 19A | | Total | Serotype19A | |  |
|  |  | n | % |  | n | % |  |
| Argentina | 419 | 14 | 3.3 | 1,267 | 56 | 4.4 | 0.3 |
| Brazil | 1,929 | 57 | 3.0 | 1,130 | 54 | 4.8 | 0.009 |
| Chile | 378 | 10 | 2.6 | 2,778 | 132 | 4.8 | 0.06 |
| Colombia | 408 | 4 | 1.0 | 788 | 20 | 2.5 | 0.06 |
| Cuba | 679 | 22 | 3.2 | 220 | 9 | 4.1 | 0.5 |
| DR^a^ | 215 | 4 | 1.9 | 329 | 10 | 3.0 | 0.4 |
| Mexico | 252 | 14 | 5.6 | 785 | 49 | 6.2 | 0.7 |
| Paraguay | 145 | 4 | 2.8 | 639 | 13 | 2.0 | 0.6 |
| Uruguay | 102 | 1 | 1.0 | 756 | 30 | 4.0 | 0.1 |
| Venezuela | 174 | 7 | 4.0 | 367 | 31 | 8.4 | 0.06 |
| **Total** | **4,701** | **137** | **2.9** | **9,059** | **404** | **4.5** | **0.009** |

^a^ Dominican Republic

References [34,82,85,86,89,97]

**Supplement 6b. *Streptococcus pneumoniae* serotype 19A. Non-meningitis and pneumoniae cases in 20 countries. SIREVA data, 2006-2007**

| Country | Non- meningitis | | | Pneumonia | | | |
| --- | --- | --- | --- | --- | --- | --- | --- |
|  | Total | Serotype 19A | | Total | | Serotype 19A | |
|  | n | n | % | n | % | n | % |
| Argentina | 213 | 17 | 8.0 | 145 | 68.1 | 10 | 6.9 |
| Bolivia ^a^ | 24 | 2 | 12.5 | 20 | 83.3 | 0 | 0.0 |
| Brazil | 159 | 7 | 4.4 | 132 | 83.0 | 6 | 4.5 |
| CAREC^b^ | 19 | 0 | 0.0 | 11 | 57.9 | 0 | 0.0 |
| Chile | 541 | 19 | 3.5 | 148 | 27.4 | 5 | 3.4 |
| Colombia | 213 | 7 | 3.3 | 99 | 46.5 | 3 | 3.0 |
| Costa Rica | 19 | 0 | 0.0 | 5 | 26.3 | 0 | 0.0 |
| Cuba | 1 | 0 | 0.0 | 1 | 100.0 | 0 | 0.0 |
| DR^c^ | 47 | 2 | 4.3 | 42 | 89.4 | 1 | 2.4 |
| Ecuador | 57 | 2 | 3.5 | 57 | 100.0 | 2 | 3.5 |
| El Salvador | 0 | 0 | 0.0 | 21 | 0.0 | 0 | 0.0 |
| Guatemala | 9 | 1 | 11.1 | 3 | 33.3 | 0 | 0.0 |
| Honduras | 0 | 0 | 0.0 | 0 | 0.0 | No data | |
| Mexico | 105 | 1 | 1.0 | 71 | 67.6 | 1 | 1.4 |
| Nicaragua | 3 | 1 | 50.0 | 3 | 100.0 | 1 | 33.3 |
| Panama | 30 | 3 | 10.0 | 10 | 33.3 | 2 | 20.0 |
| Paraguay | 139 | 3 | 2.2 | 122 | 87.8 | 3 | 2.5 |
| Peru | 35 | 1 | 2.9 | 35 | 100.0 | 0 | 0.0 |
| Uruguay | 144 | 7 | 4.9 | 125 | 86.8 | 7 | 5.6 |
| Venezuela | 43 | 2 | 4.7 | 19 | 44.2 | 1 | 5.3 |
| **Total** | **1,801** | **75** | **4.2** | **1,069** | **59.4** | **42** | **3.9** |

^a^ Only 2006

^b^Caribbean Epidemiology Center

^c^ Dominican Republic

**References [85,86]**

## **Supplement 6C. *Streptococcus pneumoniae*. Meningitis isolates by country and serotype, presented by vaccine type (VT) (PCV7, PCV10, PCV13) and non vaccine type (NVT).**

## **SIREVA data, 2000-2009^a^**

| **Country** | VT | | | | | | | | | | | | | **NVT** | **Total** |
| --- | --- | --- | --- | --- | --- | --- | --- | --- | --- | --- | --- | --- | --- | --- | --- |
|  | **PCV13** | | | | | | | | | | | | |  |  |
|  | **PCV10** | | | | | | | | | |  | | |  |  |
|  | **PCV7** | | | | | | |  | | |  |  |  |  |  |
|  | **4** | **6B** | **9V** | **14** | **18C** | **19F** | **23F** | **1** | **3** | **5** | **6A** | **7F** | **19A** |  |  |
|  | **n** | | | | | | | | | | | | | | |
| Argentina | 5 | 25 | 5 | 99 | 37 | 15 | 14 | 32 | 4 | 48 | 10 | 24 | **14** | 87 | **419** |
| Bolivia | 0 | 3 | 0 | 5 | 1 | 0 | 0 | 4 | 0 | 0 | 1 | 0 | **0** | 6 | **20** |
| Brazil | 43 | 239 | 50 | 623 | 140 | 123 | 111 | 28 | 31 | 63 | 94 | 45 | **57** | 282 | **1,929** |
| CAREC^b^ | 0 | 1 | 0 | 0 | 0 | 0 | 0 | 0 | 0 | 0 | 0 | 0 | **0** | 0 | **1** |
| Chile | 8 | 24 | 3 | 82 | 28 | 32 | 6 | 18 | 3 | 11 | 17 | 12 | **10** | 124 | **378** |
| Colombia | 3 | 48 | 9 | 101 | 34 | 22 | 40 | 26 | 6 | 21 | 25 | 8 | **4** | 61 | **408** |
| Costa Rica | 0 | 2 | 0 | 3 | 0 | 1 | 1 | 0 | 0 | 0 | 0 | 0 | **1** | 2 | **10** |
| Cuba | 7 | 93 | 29 | 80 | 102 | 93 | 35 | 60 | 17 | 17 | 24 | 48 | **22** | 52 | **679** |
| DR^c^ | 5 | 34 | 2 | 61 | 10 | 21 | 23 | 8 | 2 | 9 | 17 | 1 | **4** | 18 | **215** |
| Ecuador | 1 | 6 | 1 | 8 | 1 | 2 | 0 | 1 | 1 | 2 | 1 | 1 | **0** | 14 | **39** |
| El Salvador | 0 | 0 | 2 | 1 | 1 | 0 | 1 | 0 | 1 | 0 | 2 | 1 | **1** | 1 | **11** |
| Guatemala | 0 | 0 | 4 | 1 | 0 | 0 | 1 | 0 | 0 | 0 | 0 | 2 | **3** | 1 | **12** |
| Honduras | 0 | 2 | 0 | 1 | 0 | 0 | 1 | 0 | 0 | 0 | 0 | 0 | **0** | 4 | **8** |
| Mexico | 2 | 35 | 5 | 33 | 17 | 30 | 31 | 4 | 3 | 1 | 13 | 9 | **14** | 55 | **252** |
| Nicaragua | 0 | 0 | 0 | 0 | 0 | 0 | 1 | 0 | 0 | 1 | 0 | 0 | **0** | 1 | **3** |
| Panama | 1 | 7 | 0 | 3 | 4 | 1 | 1 | 0 | 0 | 5 | 1 | 0 | **1** | 7 | **31** |
| Paraguay | 5 | 16 | 1 | 33 | 2 | 4 | 5 | 11 | 1 | 20 | 4 | 6 | **4** | 33 | **145** |
| Peru | 0 | 6 | 0 | 6 | 1 | 8 | 2 | 1 | 1 | 2 | 0 | 0 | **1** | 4 | **32** |
| Uruguay | 1 | 8 | 0 | 20 | 4 | 2 | 1 | 6 | 2 | 21 | 5 | 10 | **1** | 21 | **102** |
| Venezuela | 3 | 29 | 2 | 49 | 14 | 11 | 9 | 8 | 1 | 19 | 6 | 8 | **7** | 8 | **174** |
| **Total** | **84** | **578** | **113** | **1,209** | **396** | **365** | **283** | **207** | **73** | **240** | **220** | **175** | **144** | **781** | **4,868** |
| **%** | **1.7** | **11.9** | **2.3** | **24.8** | **8.1** | **7.5** | **5.8** | **4.3** | **1.5** | **4.9** | **4.5** | **3.6** | **3.0** | **16.0** | **100.0** |
| **Rank** | **12** | **2** | **11** | **1** | **3** | **4** | **5** | **8** | **13** | **6** | **7** | **9** | **10** |  |  |

^a^ 2000-2005 (ten countries) and 2006-2009 (twenty countries)

^b^Caribbean Epidemiology Centre

^c^Dominican Republic

**References [34,85,86,89,97]**

## **Supplement 6d. *Streptococcus pneumoniae*. Non-meningitis isolates by country and serotype presented by vaccine type (VT) (PCV7, PCV10, PCV13) and non vaccine type (NVT).**

## **SIREVA data, 2000-2009^a^**

| Country | **VT** | | | | | | | | | | | | | **NVT** | **Total** |
| --- | --- | --- | --- | --- | --- | --- | --- | --- | --- | --- | --- | --- | --- | --- | --- |
|  | **PCV13** | | | | | | | | | | | | |  |  |
|  | **PCV10** | | | | | | | | | |  | | |  |  |
|  | **PCV7** | | | | | | |  | | |  |  |  |  |  |
|  | **4** | **6B** | **9V** | **14** | **18C** | **19F** | **23F** | **1** | **5** | **7F** | **3** | **6A** | **19A** |  |  |
|  | **n** | | | | | | | | | | | | | | |
| Argentina | 9 | 72 | 50 | 385 | 26 | 26 | 34 | 156 | 178 | 51 | 35 | 38 | **56** | 151 | **1,267** |
| Bolivia | 0 | 6 | 0 | 13 | 2 | 1 | 0 | 0 | 1 | 2 | 0 | 0 | **2** | 5 | **32** |
| Brazil | 11 | 107 | 32 | 521 | 28 | 30 | 33 | 100 | 47 | 17 | 38 | 34 | **54** | 78 | **1,130** |
| CAREC^b^ | 0 | 9 | 1 | 6 | 6 | 2 | 2 | 0 | 0 | 2 | 0 | 5 | **1** | 3 | **37** |
| Chile | 58 | 202 | 39 | 815 | 179 | 154 | 99 | 200 | 127 | 115 | 29 | 139 | **132** | 490 | **2,778** |
| Colombia | 8 | 70 | 24 | 281 | 72 | 26 | 31 | 82 | 30 | 5 | 20 | 46 | **20** | 73 | **788** |
| Costa Rica | 3 | 2 | 3 | 19 | 0 | 3 | 2 | 0 | 0 | 1 | 4 | 3 | **2** | 8 | **50** |
| Cuba | 3 | 26 | 7 | 30 | 38 | 32 | 11 | 20 | 5 | 15 | 4 | 8 | **9** | 12 | **220** |
| DR^c^ | 5 | 26 | 9 | 167 | 6 | 4 | 15 | 25 | 6 | 2 | 15 | 14 | **10** | 25 | **329** |
| Ecuador | 1 | 8 | 7 | 36 | 0 | 3 | 5 | 16 | 9 | 1 | 4 | 5 | **5** | 21 | **121** |
| El Salvador | 0 | 0 | 4 | 5 | 1 | 0 | 0 | 1 | 0 | 1 | 1 | 3 | **1** | 1 | **18** |
| Guatemala | 1 | 0 | 0 | 3 | 0 | 0 | 2 | 0 | 0 | 0 | 0 | 2 | **4** | 3 | **15** |
| Honduras | 0 | 0 | 0 | 0 | 0 | 0 | 0 | 0 | 0 | 0 | 0 | 0 | **0** | 0 | **0** |
| Mexico | 7 | 87 | 31 | 73 | 13 | 142 | 97 | 13 | 7 | 7 | 22 | 39 | **49** | 198 | **785** |
| Nicaragua | 0 | 0 | 0 | 0 | 0 | 0 | 0 | 0 | 0 | 0 | 0 | 0 | **1** | 0 | **1** |
| Panama | 1 | 12 | 2 | 14 | 3 | 5 | 3 | 1 | 5 | 1 | 1 | 4 | **7** | 3 | **62** |
| Paraguay | 9 | 36 | 25 | 255 | 3 | 7 | 14 | 63 | 92 | 17 | 4 | 12 | **13** | 89 | **639** |
| Peru | 2 | 13 | 0 | 31 | 0 | 5 | 3 | 2 | 1 | 0 | 0 | 6 | **4** | 7 | **74** |
| Uruguay | 8 | 31 | 26 | 248 | 12 | 13 | 6 | 119 | 108 | 42 | 44 | 14 | **30** | 55 | **756** |
| Venezuela | 6 | 51 | 7 | 129 | 7 | 12 | 9 | 27 | 32 | 11 | 20 | 13 | **31** | 12 | **367** |
| **Total** | **132** | **758** | **267** | **3,031** | **396** | **465** | **366** | **825** | **648** | **290** | **241** | **385** | **431** | **1,234** | **9,469** |
| **%** | **1.4** | **8.0** | **2.8** | **32.0** | **4.2** | **4.9** | **3.9** | **8.7** | **6.8** | **3.1** | **2.5** | **4.1** | **4.6** | **13.0** | **100,0** |
| **Rank** | **13** | **3** | **11** | **1** | **7** | **5** | **9** | **2** | **4** | **10** | **12** | **8** | **6** |  |  |

^a^ 2000-2005 (ten countries) and 2006-2009 (twenty countries)

^b^Caribbean Epidemiology Centre

^c^Dominican Republic

**References [34,85,86,89,97]**
