# Supplementary material for: Streptococcus pneumoniae serotype 19A in Latin America and the Caribbean: a systematic review and meta-analysis, 1990–2010
Source: BMC Infect Dis. 2012 May 28;12:124. doi: 10.1186/1471-2334-12-124 (PMC3475047; doi:10.1186/1471-2334-12-124)
Supplement: Additional file 7 — a- Streptococcus pneumoniae . Number of acute otitis media isolates presented by vaccine type (VT) (PCV7, PCV10, PCV13) and non vaccine type (NVT). b - Streptococcus pneumoniae. Number of nasopharyngeal isolates in healthy children presented by vaccine type (VT) (PCV7, PCV10, PCV13) and non vaccine type (NVT). c - Streptococcus pneumoniae. Number of nasopharyngeal isolates in sick children presented by vaccine type (VT) (PCV7, PCV10, PCV13) and non vaccine type (NVT) [35-60,76]. [file 1471-2334-12-124-S7.docx]

**Supplement 7**

**Supplement 7a. *Streptococcus pneumoniae*. Number of acute otitis media isolates arranged by vaccine type (VT) (PCV7, PCV10, PCV13) and non vaccine type (NVT)**

| **Country**  **[Ref]**  **(Year)** | **VT** | | | | | | | | | | | | | **NVT** | | | | | | **Total** |
| --- | --- | --- | --- | --- | --- | --- | --- | --- | --- | --- | --- | --- | --- | --- | --- | --- | --- | --- | --- | --- |
|  | **PCV13** | | | | | | | | | | | | |  |  |  |  |  |  |  |
|  | **PCV10** | | | | | | | | | |  | | |  |  |  |  |  |  |  |
|  | **PCV7** | | | | | | |  | | | | | |  |  |  |  |  |  |  |
|  | **4** | **6B** | **9V** | **14** | **18C** | **19F** | **23F** | **1** | **5** | **7F** | **3** | **6A** | **19A** | **10A** | **11A** | **15A** | **16F** | **18A** | **others** |  |
|  | Serotypes | | | | | | | | | | | | | | | | | | |  |
| **Brazil**  **[35]**  (1990-1995) |  | 6 | 3 |  | 3 | 9 | 3 |  |  |  |  |  | 6 |  | **3** |  | 9 |  | 6 | **48** |
| **Colombia**  **[36]**  (2008-2009) |  | 4 |  | 5 |  | 5 | 11 | 1 | 1 |  |  | 4 | 1 |  |  |  |  |  | 4 | **36** |
| **Costa Rica**  **[37]**  (1992-2007) | 5 | 30 | 10 | 46 | 5 | 101 | 23 | 1 |  | 2 | 34 | 8 | 2 | **6** | **7** | 4 | 9 | 5 | 48 | **346** |
| **Mexico**  **[76]**  (1995-2000) |  | 1 |  | 3 |  | 9 | 1 |  |  |  | 3 | 5 | 2 |  |  |  |  |  | 6 | **30** |
| **n Total**  **%** | 5 | 41 | 13 | 54 | 8 | **124** | 38 | 2 | 1 | 2 | 37 | 17 | **11** | 6 | 10 | 4 | 18 | 5 | 64 | **460** |
|  | **1.1** | **8.9** | **2.8** | **11.7** | **1.7** | **27.0** | **8.3** | **0.4** | **0.2** | **0.4** | **8.0** | **3.7** | **2.4** | **1.3** | **2.2** | **0.9** | **3.9** | **1.1** | **13.9** |  |
| **Rank** | **13** | **3** | **8** | **2** | **11** | **1** | **4** | **15** | **16** | **15** | **5** | **7** | **9** | **12** | **10** | **14** | **6** | **13** |  |  |

Note: 9 serotypes 16F were 16*

**Supplement 7b. *Streptococcus pneumoniae*. Number of nasopharyngeal isolates in healthy children arranged by vaccine type (VT) (PCV7, PCV10, PCV13) and non vaccine type (NVT)**

| **Country**  **[Ref]**  **(Year)** | **VT** | | | | | | | | | | | | | **NVT** | | | | | | | | | | **Total** |
| --- | --- | --- | --- | --- | --- | --- | --- | --- | --- | --- | --- | --- | --- | --- | --- | --- | --- | --- | --- | --- | --- | --- | --- | --- |
|  | **PCV13** | | | | | | | | | | | | |  |  |  |  |  |  |  |  |  |  |  |
|  | **PCV10** | | | | | | | | | |  | | |  |  |  |  |  |  |  |  |  |  |  |
|  | **PCV7** | | | | | | |  | | |  |  |  |  |  |  |  |  |  |  |  |  |  |  |
|  | **4** | **6B** | **9V** | **14** | **18C** | **19F** | **23F** | **1** | **5** | **7F** | **3** | **6A** | **19A** | **10F** | **11A** | **13** | **15B** | **15C** | **22F** | **23B** | **35B** | **NT^a^** | **O^b^** |  |
|  | **n** | | | | | | | | | | | | | | | | | | | | | | | |
| **Argentina**  **[39]**  **(2000-2007)** |  | 53 | 43 | 56 |  | 62 | 51 |  |  |  |  | 87 | 27 |  | 26 |  | 72 | 26 |  |  |  |  | 225 | **728** |
| **Brazil**  **[40]**  **(1998)** |  | 14 | 9 | 7 | 10 | 20 | 10 |  | 1 |  | 2 | 15 | 1 |  | 4 | 1 | 5 | 3 | 4 | 1 | 1 | 2 | 19 | **135** |
| **Brazil**  **[41]**  **(2000)** | 2 | 29 | 8 | 33 | 11 | 19 | 14 | 1 | 2 | 5 | 3 | 24 | 10 | 1 | 7 |  | 6 |  | 2 | 1 |  |  | 30 | **222** |
| **Brazil**  **[42]**  **(2000-2001)** |  | 1 |  | 1 | 1 | 6 | 3 |  |  |  |  | 5 | 1 |  |  |  | 1 |  | 1 | 3 |  | 3 | 6 | **33** |
| **Brazil**  **[43]**  **(2005)** | 5 | 20 | 9 | 112 | 10 | 13 | 25 | 1 | 1 | 1 |  | 22 | 21 |  | 8 | 1 | 1 | 1 | 3 | 9 |  | 35 | 34 | **332** |
| **Chile**  **[44]**  **(1994-1999)** |  | 3 |  | 14 |  | 43 | 7 |  |  |  |  | 1 | 0 |  |  |  |  |  |  |  |  |  |  | **68** |
| **Chile**  **[45]**  **(1994-1995)** |  | 4 |  | 0 |  | 7 | 3 | 1 |  | 1 |  | 9 | 5 |  |  |  | 8 | 7 |  |  |  |  | 5 | **50** |
| **Chile**  **[46]**  **(2001-2003)** | 4 | 50 | 5 | 56 | 10 | 61 | 59 | 3 |  | 12 | 7 | 75 | 16 |  |  |  |  |  |  |  |  | 121 | 235 | **714** |
| **Colombia**  **[47]**  **(2009)** | 3 |  | 3 | 22 |  | 16 | 20 | 3 |  |  | 2 | 33 | 6 |  |  |  |  |  |  |  |  |  | 62 | **170** |
| **Mexico**  **[48]**  **(1994)** | 2 | 12 |  | 4 | 3 | 7 | 3 |  |  | 1 | 2 | 3 | 4 |  | 1 |  |  |  |  |  |  |  | 21 | **65** |
| **Mexico**  **[38]**  **(1997-2000)** | 1 | 24 | 2 | 6 | 3 | 65 | 7 | 1 | 1 |  | 1 | 35 | 6 |  | 2 | 1 |  |  |  |  |  |  | 18 | **173** |
| **Mexico**  **[49]**  **(2002)** | 2 | 129 | 8 | 33 | 14 | 191 | 93 |  |  |  | 2 | 124 | 50 |  | 34 | 31 |  |  | 20 | 3 | 32 |  | 60 | **829** |
| **Mexico**  **[50]**  **(2002-2003)** |  |  |  | 4 | 6 | 17 | 19 |  |  |  |  | 7 | 0 | 7 | 18 |  |  |  |  |  |  |  | 51 | **122** |
| **Mexico**  **[51]**  **(2006)** |  | 47 | 1 | 6 |  | 8 | 9 |  |  | 1 | 3 | 14 | 19 | 2 | 14 |  | 15 |  | 1 | 5 | 4 |  | 25 | **176** |
| **Panama**  **[52]**  **(2008)** | 1 | 35 | 1 | 12 |  | 32 | 11 |  |  |  | 6 | 14 | 2 |  | 20 |  | 5 | 4 |  |  |  | 3 | 16 | **163** |
| **Peru**  **[53]**  **(2000)** | 2 | 15 | 3 | 11 | 1 | 12 | 20 |  | 1 |  |  | 15 | 3 | 1 | 2 | 1 | 13 | 1 |  | 4 |  | 13 | 27 | **146** |
| **Uruguay**  **[54]**  **(1993-1995)** |  | 3 | 2 | 13 |  | 5 | 6 | 1 |  |  | 4 | 15 | 3 |  |  |  |  |  |  |  |  |  | 22 | **74** |
| **Venezuela**  **[55]**  **(2004-2005)** |  | 14 | 4 | 7 | 2 | 11 |  |  |  |  |  | 30 | 5 |  | 11 |  | 16 |  |  |  |  |  | 19 | **122** |
| **Venezuela**  **[56]**  **(2008)** |  | 30 |  | 0 |  |  | 1 |  |  |  |  | 2 | 1 |  |  |  |  |  |  |  |  |  | 24 | **58** |
| **n Total** | **22** | **483** | **98** | **397** | **71** | **595** | **361** | **11** | **6** | **21** | **32** | **530** | **180** | **11** | **147** | **35** | **142** | **42** | **31** | **26** | **37** | **177** | **896** | **4380** |
| **%** | **0.5** | **11.0** | **2.2** | **9.1** | **1.6** | **13.6** | **8.2** | **0.3** | **0.1** | **0.5** | **0.7** | **12.1** | **4.1** | **0.3** | **3.4** | **0.8** | **3.2** | **1.0** | **0.7** | **0.6** | **0.8** | **4.1** | **20.5** |  |
| **Rank** | **15** | **3** | **9** | **4** | **10** | **1** | **5** | **16** | **17** | **15** | **13** | **2** | **6** | **16** | **7** | **12** | **8** | **11** | **13** | **14** | **12** | **6** |  |  |

^a^Non typable

^b^Others: 44 different serotypes

Note: 36 isolates serotype 6 were included in 6A were 6*, 3 serotypes 10 were included in 10A and 20 serotype 11 in 11A

**Supplement 7c. *Streptococcus pneumoniae*. Number of nasopharyngeal isolates in sick children arranged by vaccine type (VT) (PCV7, PCV10, PCV13) and non vaccine type (NVT)**

| **Country** | **VT** | | | | | | | | | | | | | **NT** | | | | | | Total |
| --- | --- | --- | --- | --- | --- | --- | --- | --- | --- | --- | --- | --- | --- | --- | --- | --- | --- | --- | --- | --- |
|  | **PCV13** | | | | | | | | | | | | |  |  |  |  |  |  |  |
|  | **PCV10** | | | | | | | | | |  | | |  |  |  |  |  |  |  |
|  | **PCV7** | | | | | | |  | | | | | |  |  |  |  |  |  |  |
|  | **4** | **6B** | **9V** | **14** | **18C** | **19F** | **23F** | **1** | **5** | **7F** | **3** | **6A** | **19A** | **10A** | **11A** | **15B** | **20** | **NT^a^** | **O^b^** |  |
|  | n | | | | | | | | | | | | | | | | | | | |
| Brazil  **[57]**  **(1997-2001)** | 3 | 20 | 6 | 20 | 10 | 15 | 21 | 3 | 5 |  |  | 16 | **3** | 5 | 10 | 6 | 8 | 4 | 7 | **162** |
| Brazil  **[58]**  **(2002-2003)** |  | 1 |  |  | 2 | 1 | 2 |  |  |  |  | 1 | **1)** | 1 |  |  |  | 2 | 1 | **12** |
| Brazil  **[59]**  **(2009)** | 1 | 10 |  | 14 |  | 11 | 8 |  |  |  | 2 | 9 | **7** |  | 3 | 3 |  |  | 3 | **71** |
| Mexico  **[48]**  **(1994)** | 3 | 10 |  | 3 | 2 | 9 | 4 |  |  | 1 | 2 | 3 | **5** | 2 |  |  |  |  | 25 | **69** |
| Uruguay  **[54]**  **(1993-1995)** |  | 2 | 1 | 4 |  | 3 | 4 | 1 |  |  |  | 1 | **0** |  |  |  |  |  | 5 | **21** |
| **Venezuela**  **[60]**  **(2000)** |  | 6 | 1 | 4 |  | 3 | 6 |  |  |  |  | 3 | **0** |  |  | 1 |  | 3 |  | **27** |
| **Total n**  **%** | **7** | **49** | **8** | **45** | **14** | **42** | **45** | **4** | **5** | **1** | **4** | **33** | **16** | **8** | **13** | **10** | **8** | **9** | **41** | **362** |
|  | **1.9** | **13.5** | **2.2** | **12.4** | **3.9** | **11.6** | **12.4** | **1.1** | **1.4** | **0.3** | **1.1** | **9.1** | **4.4** | **2.2** | **3.6** | **2.8** | **2.2** | **2.5** | **12.7** |  |
| Rank | **14** | **1** | **11** | **2** | **7** | **4** | **2** | **16** | **15** | **18** | **16** | **5** | **6** | **11** | **8** | **9** | **11** | **10** |  |  |

^a^ Non typable

^b^Others: 7 different serotypes
