# Supplementary material for: Streptococcus pneumoniae serotype 19A in Latin America and the Caribbean: a systematic review and meta-analysis, 1990–2010
Source: BMC Infect Dis. 2012 May 28;12:124. doi: 10.1186/1471-2334-12-124 (PMC3475047; doi:10.1186/1471-2334-12-124)
Supplement: Additional file 8 — a- Streptococcus pneumoniae . Meningitis and non-meningitis isolates, penicillin resistance by serotype. SIREVA data 2007–2009. b - Streptococcus pneumoniae serotype 19A. Meningitis isolates, penicillin resistance. Argentina, Brazil, Colombia, Mexico and Venezuela. 2007–2009 [86,89,97]. [file 1471-2334-12-124-S8.docx]

**Supplement 8**

**Supplement 8a. *Streptococcus pneumoniae*. Meningitis and non-meningitis isolates, penicillin resistance by serotype. All countries, SIREVA data 2007-2009**

| **Serotypes** | **Meningitis** | | | **Non-meningitis** | | | | |
| --- | --- | --- | --- | --- | --- | --- | --- | --- |
|  | **PRSP**  **Resistance**^a^ | | **Total**  **serotype** | **PRSP** | | | | **Total serotype** |
|  |  |  |  | **Intermediate^b^** | | **Resistance^b^** | |  |
|  | **n** | **%** |  | **n** | **%** | **n** | **%** |  |
| 1 | 1 | 1.9 | 52 | 0 | 0.0 | 1 | 0.5 | 216 |
| 3 | 0 | 0.0 | 27 | 0 | 0.0 | 0 | 0.0 | 75 |
| 4 | 0 | 0.0 | 18 | 0 | 0.0 | 0 | 0.0 | 32 |
| 5 | 0 | 0.0 | 36 | 0 | 0.0 | 0 | 0.0 | 147 |
| 6A | 23 | 32.9 | 70 | 2 | 1.3 | 1 | 0.7 | 151 |
| 6B | 69 | 46.6 | 148 | 9 | 3.9 | 2 | 0.9 | 232 |
| 7F | 0 | 0.0 | 46 | 0 | 0.0 | 0 | 0.0 | 91 |
| 9V | 7 | 41.2 | 17 | 6 | 8.5 | 2 | 2.8 | 71 |
| 14 | 227 | 70.9 | 320 | 120 | 11.9 | 16 | 1.6 | 1008 |
| 18C | 3 | 3.7 | 82 | 0 | 0.0 | 1 | 1.4 | 73 |
| 19A | 22 | 53.7 | 41 | 17 | 10.8 | 5 | 3.2 | 158 |
| 19F | 37 | 48.7 | 76 | 27 | 18.9 | 11 | 7.7 | 143 |
| 23F | 41 | 77.4 | 53 | 9 | 7.5 | 5 | 4.2 | 120 |
| Others | 23 | 12.8 | 180 | 14 | 3.6 | 0 | 0.0 | 387 |
| Total | 453 | 38.9 | 1166 | 204 | 7.0 | 44 | 1.5 | 2904 |

Clinical and Laboratory Standards Institute. Performance Standards for Antimicrobial Susceptibility Testing. Twentieth informational supplement CLSI document M100-S20; 30. Wayne, PA: Clinical and Laboratory Standards Institute; 2010.

^a^Penicillin resistant ≥ 0.125 µg/ml

^b^Penicillin intermediate resistance (IR)= 4.0 µg/ml , high resistance (HR) ≥ 8.0 µg

**References [86,89,97]**

**Supplement 8a. *Streptococcus pneumoniae*. Meningitis and non-meningitis isolates, penicillin resistance (high and intermediate) by serotype. All countries, SIREVA data 2007-2009**

| **Serotypes** | **Meningitis (n=1166)** | | **Non-meningitis (n=2904)** | | | |
| --- | --- | --- | --- | --- | --- | --- |
|  | **Resistant**  **(n=453)** | | **Intermediate**  **(n=204)** | | **Resistant**  **(n=44)** | |
|  | **n** | **%** | **n** | **%** | **n** | **%** |
| 1 | 1 | 0.2 | 0 | 0.0 | 1 | 2.3 |
| 3 | 0 | 0.0 | 0 | 0.0 | 0 | 0.0 |
| 4 | 0 | 0.0 | 0 | 0.0 | 0 | 0.0 |
| 5 | 0 | 0.0 | 0 | 0.0 | 0 | 0.0 |
| 6A | 23 | 5.1 | 2 | 1.0 | 1 | 2.3 |
| 6B | 69 | 15.2 | 9 | 4.4 | 2 | 4.5 |
| 7F | 0 | 0.0 | 0 | 0.0 | 0 | 0.0 |
| 9V | 7 | 1.5 | 6 | 2.9 | 2 | 4.5 |
| 14 | 227 | 50.1 | 120 | 58.8 | 16 | 36.4 |
| 18C | 3 | 0.7 | 0 | 0.0 | 1 | 2.3 |
| 19A | 22 | 4.9 | 17 | 8.3 | 5 | 11.4 |
| 19F | 37 | 8.2 | 27 | 13.2 | 11 | 25.0 |
| 23F | 41 | 9.1 | 9 | 4.4 | 5 | 11.4 |
| Others | 23 | 5.1 | 14 | 6.9 | 0 | 0.0 |
| Total | 453 | 38.9 | 204 | 7.0 | 44 | 1.5 |

Clinical and Laboratory Standards Institute. Performance Standards for Antimicrobial Susceptibility Testing. Twentieth informational supplement CLSI document M100-S20; 30. Wayne, PA: Clinical and Laboratory Standards Institute; 2010.

^a^Penicillin resistant ≥ 0.125 µg/ml

^b^Penicillin intermediate resistance (IR)= 4.0 µg/ml , high resistance (HR) ≥ 8.0 µg

**References [86,89,97]**

**Supplement 8b. *Streptococcus pneumoniae* serotype 19A. Meningitis isolates, penicillin resistance. Argentina, Brazil, Colombia, Mexico and Venezuela. 2007-2009**

| **Meningitis** | | | | | | |
| --- | --- | --- | --- | --- | --- | --- |
| **Country** | **Serotype 19A** | | | **Total serotypes** | | |
|  | **R** | **total** | **R/total** | **R** | **total** | **R/total** |
|  | **n** | **n** | **%** | **n** | **n** | **%** |
| Argentina | 5 | 6 | 83.3 | 30 | 138 | 23.4 |
| Brazil | 10 | 16 | 62.5 | 237 | 482 | 49.2 |
| Colombia | 1 | 2 | 50.0 | 28 | 88 | 31.8 |
| Mexico | 2 | 2 | 100.0 | 31 | 41 | 75.6 |
| Venezuela | 0 | 4 | 0.0 | 14 | 44 | 35.9 |
| Total | 18 | 30 | 60.0 | 340 | 793 | 42.9 |

| **Meningitis** | | | |
| --- | --- | --- | --- |
| **Country** | **R 19A** | **Total R** | **R 19A/total R** |
|  | n | n | % |
| Argentina | 5 | 30 | 16.7 |
| Brazil | 10 | 237 | 4.2 |
| Colombia | 1 | 28 | 3.6 |
| Mexico | 2 | 31 | 6.5 |
| Venezuela | 0 | 14 | 0 |
| Total | 18 | 340 | 5.3 |

**Supplement 8b. *Streptococcus pneumoniae* serotype 19A. Non-meningitis isolates, penicillin resistance. Argentina, Brazil, Colombia, Mexico and Venezuela. 2007-2009**

| Non meningitis | | | | | | | | | | | |
| --- | --- | --- | --- | --- | --- | --- | --- | --- | --- | --- | --- |
| Country | Serotype 19A | | | Total | | | Total serotypes | | | | |
|  | I | R | total | | I/Total | R/total | I | R | total | I/Total | R/total |
|  | n | | | | % | | n | | | % | |
| Argentina | 0 | 0 | 21 | | 0.0 | 0.0 | 1 | 0 | 430 | 0.2 | 0.0 |
| Brazil | 3 | 0 | 13 | | 23.1 | 0.0 | 53 | 0 | 320 | 16,6 | 0.0 |
| Colombia | 2 | 1 | 11 | | 18.2 | 9.1 | 51 | 2 | 320 | 15.9 | 0.6 |
| Mexico | 4 | 2 | 19 | | 21.5 | 10.5 | 42 | 23 | 252 | 16.7 | 9.1 |
| Venezuela | 3 | 0 | 9 | | 33.3 | 0.0 | 3 | 0 | 59 | 5.1 | 0.0 |
| Total | 12 | 3 | 73 | | 16.4 | 4.1 | 150 | 25 | 1381 | 10.9 | 1.8 |

| Non-meningitis | | | | | | |
| --- | --- | --- | --- | --- | --- | --- |
| Country | R 19A | | R Total | | R 19A/R total | |
|  | I | R | I | R | I | R |
|  | n | | n | | % | |
| Argentina | 0 | 0 | 1 | 0 | 0 | 0 |
| Brazil | 3 | 0 | 53 | 0 | 5.7 | 0 |
| Colombia | 2 | 1 | 51 | 2 | 3.9 | 50.0 |
| Mexico | 4 | 2 | 42 | 23 | 9.5 | 8.7 |
| Venezuela | 3 | 0 | 3 | 0 | 100 | 0 |
| Total | 12 | 3 | 150 | 25 | 8.0 | 12.0 |
