# Supplementary material for: Streptococcus pneumoniae serotype 19A in Latin America and the Caribbean: a systematic review and meta-analysis, 1990–2010
Source: BMC Infect Dis. 2012 May 28;12:124. doi: 10.1186/1471-2334-12-124 (PMC3475047; doi:10.1186/1471-2334-12-124)
Supplement: Additional file 9 — Pneumococcal diseases in Latin America and the Caribbean incidences by country [[27],[29],[33],[46],[77]-[79],[83],[134],[135]]. [file 1471-2334-12-124-S9.docx]

## **Supplement 9**

## **Pneumococcal diseases in Latin America and the Caribbean incidences by country**

| **Country** | **Study period** | | **Disease** | | **Age (months)** | **Incidence**  **(Cases per 100,000 )** | **[Ref]** |
| --- | --- | --- | --- | --- | --- | --- | --- |
| Argentina | 1999-2002 | | IPD | | 2-5 | 114.9 | [27] |
|  |  |  |  |  | 6-11 | 246.7 |  |
|  |  |  |  |  | 12-17 | 238.2 |  |
|  |  |  |  |  | 18-23 | 199.2 |  |
|  |  |  |  |  | **2-23** | 206.8 |  |
|  |  |  | Meningitis | | 2-5 | 19.1 |  |
|  |  |  |  |  | 6-11 | 4.3 |  |
|  |  |  |  |  | 12 - 17 | 8.5 |  |
|  |  |  |  |  | 18-23 | 4.3 |  |
|  |  |  |  |  | **2-23** | 8.1 |  |
|  | 1999-2003 | | Pneumonia | | 2-5 | 6.4 |  |
|  |  |  |  |  | 6-11 | 76.6 |  |
|  |  |  |  |  | 12 - 17 | 136.1 |  |
|  |  |  |  |  | 18 - 23 | 131.9 |  |
|  |  |  |  |  | **2- 23** | 95.9 |  |
|  | 1999-2004 | | Bacteremia | | 2 - 5 | 83 |  |
|  |  |  |  |  | 6 - 11 | 161.6 |  |
|  |  |  |  |  | 12-17 | 85.1 |  |
|  |  |  |  |  | 18-23 | 59.6 |  |
|  |  |  |  |  | **2-23** | **98.6** |  |
| Brazil | 1995-1999 | | Meningitis | | 0-60 | 24.7 | [29] |
| Brazil | 1997-1998 | | Meningitis (All causes) | | 0-12 | 334.9 | [77] |
|  |  |  |  |  | 0-60 | 115 |  |
| Chile | 1994-1999 | | IPD  (Hospitalized) | | 0-5 | 61.0 | [134] |
|  |  |  |  |  | 6-11 | 49.3 |  |
|  |  |  |  |  | 12-23 | 26.9 |  |
|  |  |  |  |  | 24-35 | 10.0 |  |
|  |  |  |  |  | **0-35** | **30.7** |  |
|  | 1999-2001 | | IPD  (Hospitalized) | | 0-5 | 51.1 |  |
|  |  |  |  |  | 6- 11 | 61.5 |  |
|  |  |  |  |  | 12-23 | 32.9 |  |
|  |  |  |  |  | 24-35 | 12.6 |  |
|  |  |  |  |  | **0-35** | **33.9** |  |
|  | 1999-2001 | | IPD  (Emergency Room) | | 0-5 | 1.7 |  |
|  |  |  |  |  | 6-11 | 63.3 |  |
|  |  |  |  |  | 12-23 | 36. |  |
|  |  |  |  |  | 24-35 | 12.1 |  |
|  |  |  |  |  | **0-35** | **27.0** |  |
| Chile | 1994-2007 | | IPD  (Hospitalized) | | 0-5 | 58.6 | [46] |
|  |  |  |  |  | 6-35 | 32.0 |  |
|  |  |  |  |  | 36-59 | 8.2 |  |
|  |  |  |  |  | **0-60** | **10.4** |  |
| Costa Rica | 1995-2001 | | IPD | | 0-12 | 10.9 | [79] |
|  |  |  |  |  | 12-24 | 1.3 |  |
|  |  |  |  |  | **0-60** | **2.92** |  |
| Colombia | | 2008 | Bacteremic Pneumonia | 1-36 | | 41.03 | [33] |
|  |  |  | Bacteremia | 1-36 | | 15.39 |  |
|  |  |  | Meningitis | **1-36** | | **2.56** |  |
|  |  |  | Sepsis | **1-36** | | **2.56** |  |
|  |  |  | Pneumonia  (radiologically confirmed) | 1-5 | | 1397.1 |  |
|  |  |  |  | 6-11 | | 1902.4 |  |
|  |  |  |  | 12-23 | | 1598.2 |  |
|  |  |  |  | **24-36** | | 100.8 |  |
|  |  |  |  | **1-36** | | 1420.6 |  |
| Cuba | | 1998 | Meningitis (All causes) | 12-60 | | 6.5 | [135] |
|  |  | 1999 |  |  |  | 8.5 |  |
|  |  | 2000 |  |  |  | 5.8 |  |
|  |  | 2001 |  |  |  | 4.6 |  |
|  |  | 2002 |  |  |  | 3.4 |  |
|  |  | 2003 |  |  |  | 4.2 |  |
|  |  | 1998 | Meningitis | 12-60 | | 0.9 |  |
|  |  | 1999 |  |  |  | 1.3 |  |
|  |  | 2000 |  |  |  | 1.4 |  |
|  |  | 2001 |  |  |  | 0.9 |  |
|  |  | 2002 |  |  |  | 0.7 |  |
|  |  | 2003 |  |  |  | 1.0 |  |
| Guatemala | | 1996-1999 | Meningitis (All causes) | 0-60 | | 85.4 | [78] |
|  |  |  | Meningitis |  |  | 11.7 |  |
| Uruguay | | 2001-2002 | Consolidated pneumonia  (All causes) | 0-60 | | 1109 | [83] |
|  |  | 2002-200 |  |  |  | 1263 |  |
|  |  | 2003-2004 |  |  |  | 1152 |  |
|  |  | 2001-2002 |  | 0-11 | | 1692 |  |
|  |  | 2002-2003 |  |  |  | 1755 |  |
|  |  | 2003-2004 |  |  |  | 1839 |  |
|  |  | 2001-2002 |  | 12-23 | | 1757 |  |
|  |  | 2002-2003 |  |  |  | 2017 |  |
|  |  | 2003-2005 |  |  |  | 1996 |  |
|  |  | 2001-2002 |  | 24-35 | | 995 |  |
|  |  | 2002-2003 |  |  |  | 1057 |  |
|  |  | 2003-2006 |  |  |  | 850 |  |
|  |  | 2001-2002 |  | 36-59 | | 542 |  |
|  |  | 2002-2003 |  |  |  | 737 |  |
|  |  | 2003-2007 |  |  |  | 531 |  |
