# Supplementary material for: Streptococcus pneumoniae serotype 19A in Latin America and the Caribbean: a systematic review and meta-analysis, 1990–2010
Source: BMC Infect Dis. 2012 May 28;12:124. doi: 10.1186/1471-2334-12-124 (PMC3475047; doi:10.1186/1471-2334-12-124)
Supplement: Additional file 10 — Pneumococcal vaccines. Hypothetical vaccine impact, population <6 years [27-30,32-36,40-42,46,49,50,55,56,58,74,75,82,85-90,97,108,112,113,117,119,123,129]. [file 1471-2334-12-124-S10.docx]

**Supplement 10**

**Pneumococcal vaccines. Hypothetical vaccine impact, population <6 years**

| **Country** | **Year** | **Hypothetical vaccine impact (%)** | | | | | | **Comments** | **[Ref]** |
| --- | --- | --- | --- | --- | --- | --- | --- | --- | --- |
|  |  | **Vaccine valency** | | | | | |  |  |
|  |  | **7** | **9** | **10** | **11** | **13** | **23** |  |  |
| Mexico | 1995 |  |  |  |  |  | 75.8 | Invasive | [32] |
| Brazil | 1996 |  |  |  |  |  | 82.7 | Invasive | [28] |
| Brazil | 1997 | 77.7 |  |  |  |  |  | Invasive | [29] |
| Mexico | 1997 | 60.0 |  |  |  |  |  | Invasive | [88] |
| Chile | 1998 |  | 69.0 | 76.0 |  |  |  | Invasive | [90] |
| Uruguay | 2000 |  |  |  |  |  | 96.0 | Invasive | [74] |
| Uruguay | 2000 | 77.6 |  |  |  |  |  | Invasive | [108] |
| Latin America | 2001 | 58.0 | 76.2 | 79.3^a^ | 80.7 | 84.5^a^  (3.3)^b^ |  | Invasive | [75] |
| Brazil | 2001 | 50.0 |  |  |  |  |  | AOM | [35] |
| Argentina | 2002 | 53.3 | 77.4 |  | 82.6 |  |  | Invasive | [112] |
| Uruguay | 2003 | 49.0 | 76.0 |  | 86.0 |  |  | Invasive | [113] |
| Brazil | 2003 | 63.0 |  |  |  |  |  | NP^bc^ | [40] |
| Brazil | 2005 |  |  |  |  |  | 53.8 | NP^c^ | [58] |
| Peru | 2005 | 65.0 |  |  |  |  |  | Invasive | [117] |
| Mexico | 2005 | 54.0 |  |  |  |  |  | Invasive | [50] |
| Colombia | 2006 | 64.7 |  | 80.7 |  | 89.3  (0.4)^b^ |  | Invasive | [119] |
| Brazil | 2006 | 52.2 |  |  |  |  |  | NP^c^ | [41] |
|  |  | 62.4 |  |  |  |  |  | Invasive |  |
| Argentina | 2006 | 72.8 |  |  |  |  |  | Invasive | [27] |
| Argentina | 2006 | 73.0 | 91.0 |  | 95.0 |  |  | Invasive | [123] |
| Mexico | 2007 | 56.0 |  |  | 56.7 | 77.9  (6.0)^b^ |  | Invasive | [49] |
| Chile | 2007 | 40.0 to 60.0 |  |  |  |  |  | Invasive <2 years | [30] |
|  |  | 40.0 to 63.0 |  |  |  |  |  | Invasive 2 - 4 years |  |
| Venezuela | 2008 | 65.0 |  |  |  |  |  | Invasive | [55] |
| Uruguay | 2008 | 60.0 |  | 83.8 |  |  |  | Invasive <2 years | [129] |
|  |  | 38.2 |  | 88.6 |  |  |  | Invasive 2- <5 years |  |
| Chile | 2008 | 54.4 |  | 72.4 |  | 82.5  (4.2)^b^ |  | Invasive | [46] |
| Brazil | 2008 | 77.0 |  |  |  |  |  | VIH | [87] |
| Brazil | 2008 | 51.0 |  |  |  |  |  | NP^b^ | [42] |
| Colombia | 2008 | 79.2 |  |  |  |  |  | Invasive | [33] |
| Colombia | 2009 | 46.7 |  |  |  |  |  | AOM^c^ | [36] |
| Latin America, Caribe | 2009 | 64.2 |  | 80.4 |  | 89.5  (3.7)^b^ |  | Invasive | [34] |
| Venezuela | 2010 | 58.0 |  |  |  |  |  | NP^c^ | [56] |
| LAC  SIREVA | 2000-2005 | 58.3 |  | 75.3 |  | 84.7  (3.6)^b^ |  | Invasive | [82] |
|  |  | 63.4 |  | 81.6 |  | 91.8  (3.8) ^b^ |  | Pneumoniae | [34]^e^ |
|  |  | 65.1 |  | 79.2 |  | 87.4  (2.8) ^b^ |  | Meningitis | [34]^e^ |
|  | 2006-2009 | 59.2 |  | 74.5 |  | 86.6  (4.6) ^b^ |  | Invasive | [85,86,89,97]^f^ |
|  | 2006-2009 | 61.7 |  | 73.4 |  | 83.9  (3.3) ^b^ |  | Meningitis | [85,86,89,97]^f^ |
|  | 2006-2009 | 58.2 |  | 74.8 |  | 87.7  (5.2) ^b^ |  | Non-meningitis | [85,86,89,97]^f^ |

^a^ Calculated from data in reference [75]

^b^ %Spn19A

^c^ NP nasopharyngeal

^d^ AOM acute otitis media

^e^ Ten countries [34]

^f^ Twenty countries [85,86,89,97]
