# Supplementary material for: Streptococcus pneumoniae serotype 19A in Latin America and the Caribbean: a systematic review and meta-analysis, 1990–2010
Source: BMC Infect Dis. 2012 May 28;12:124. doi: 10.1186/1471-2334-12-124 (PMC3475047; doi:10.1186/1471-2334-12-124)
Supplement: Additional file 11 — Streptococcus pneumoniae serotype 19A prevalence in the region. SIREVA data 2000–2009. [file 1471-2334-12-124-S11.ppt]

## Slide 1
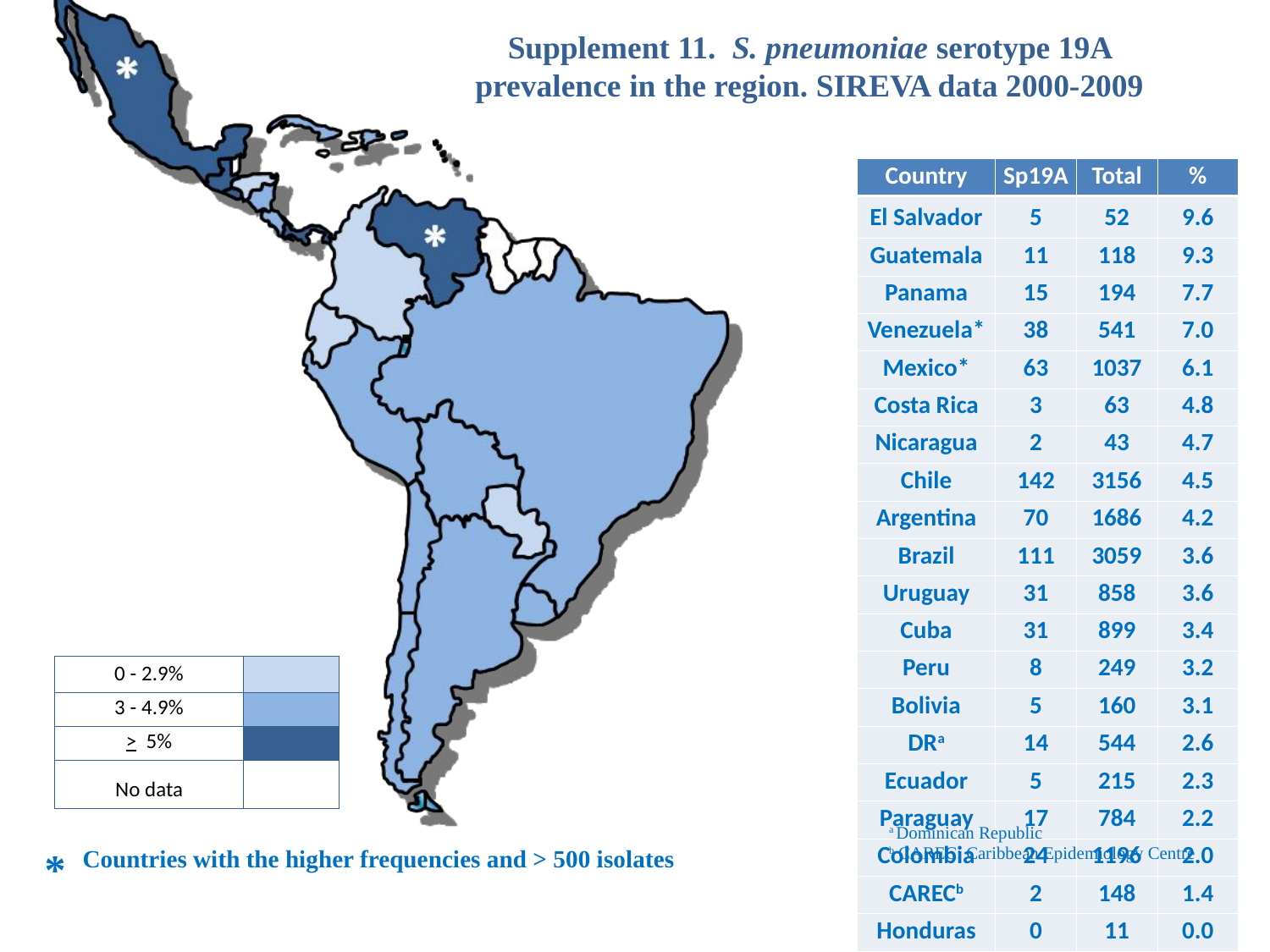

Supplement 11. S. pneumoniae serotype 19A prevalence in the region. SIREVA data 2000-2009
| Country | Sp19A | Total | % |
| --- | --- | --- | --- |
| El Salvador | 5 | 52 | 9.6 |
| Guatemala | 11 | 118 | 9.3 |
| Panama | 15 | 194 | 7.7 |
| Venezuela\* | 38 | 541 | 7.0 |
| Mexico\* | 63 | 1037 | 6.1 |
| Costa Rica | 3 | 63 | 4.8 |
| Nicaragua | 2 | 43 | 4.7 |
| Chile | 142 | 3156 | 4.5 |
| Argentina | 70 | 1686 | 4.2 |
| Brazil | 111 | 3059 | 3.6 |
| Uruguay | 31 | 858 | 3.6 |
| Cuba | 31 | 899 | 3.4 |
| Peru | 8 | 249 | 3.2 |
| Bolivia | 5 | 160 | 3.1 |
| DRa | 14 | 544 | 2.6 |
| Ecuador | 5 | 215 | 2.3 |
| Paraguay | 17 | 784 | 2.2 |
| Colombia | 24 | 1196 | 2.0 |
| CARECb | 2 | 148 | 1.4 |
| Honduras | 0 | 11 | 0.0 |
| 0 - 2.9% | |
| --- | --- |
| 3 - 4.9% | |
| > 5% | |
| No data | |
a Dominican Republic
b CAREC: Caribbean Epidemiology Centre
*
Countries with the higher frequencies and > 500 isolates
